# Supplementary material for: Air- and water-stable and photocatalytically active germanium-based 2D perovskites by organic spacer engineering
Source: Cell Rep Phys Sci. 2023 Jan 18;4(1):101214. doi: 10.1016/j.xcrp.2022.101214 (PMC10246422; doi:10.1016/j.xcrp.2022.101214)
Supplement: Document S1. Figures S1–S9, Tables S1–S4, and Note S1 [file mmc1.pdf]

Cell Reports Physical Science, Volume *4*

Supplemental information

Air- and water-stable and photocatalytically active germanium-based 2D perovskites by organic spacer engineering

Lidia Romani, Andrea Speltini, Rossella Chiara, Marta Morana, Clarissa Coccia, Costanza Tedesco, Vincenza Armenise, Silvia Colella, Antonella Milella, Andrea Listorti, Antonella Profumo, Francesco Ambrosio, Edoardo Mosconi, Riccardo Pau, Federico Pitzalis, Angelica Simbula, Damiano Ricciarelli, Michele Saba, Maria Medina-Llamas, Filippo De Angelis, and Lorenzo Malavasi

SUPPLEMENTAL INFORMATION

(a)

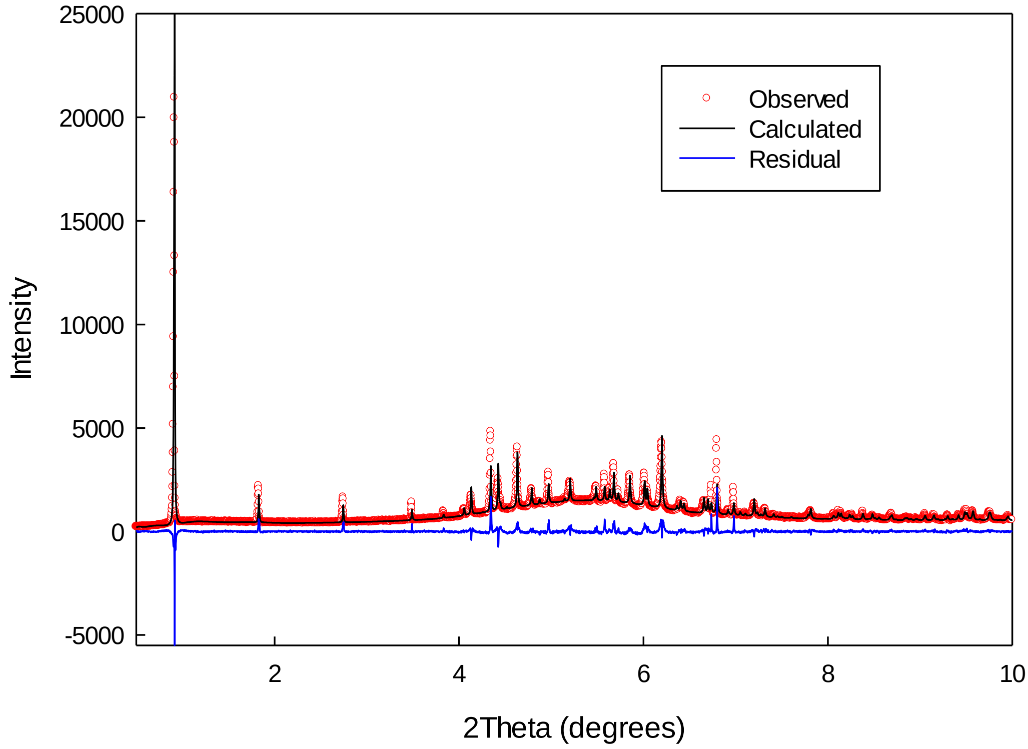

(b)

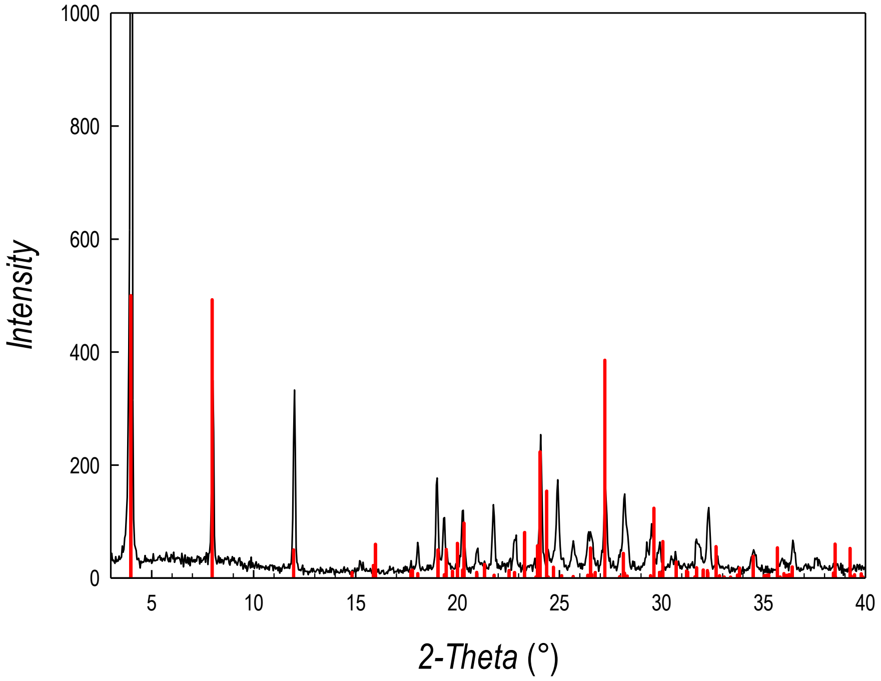

Figure S1. Structural properties of $PhBz_2GeBr_4$. Related to Figure 1. a) Indexed patter of $PhBz_2GeBr_4$ from synchrotron data (λ=0.3547); b) Diffraction pattern (Cu-alpha radiation) of $PhBz_2GeBr_4$ against the expected Bragg peaks of $BPEA_2PbI_4$ with actual lattice parameters obtained from the indexing procedure.

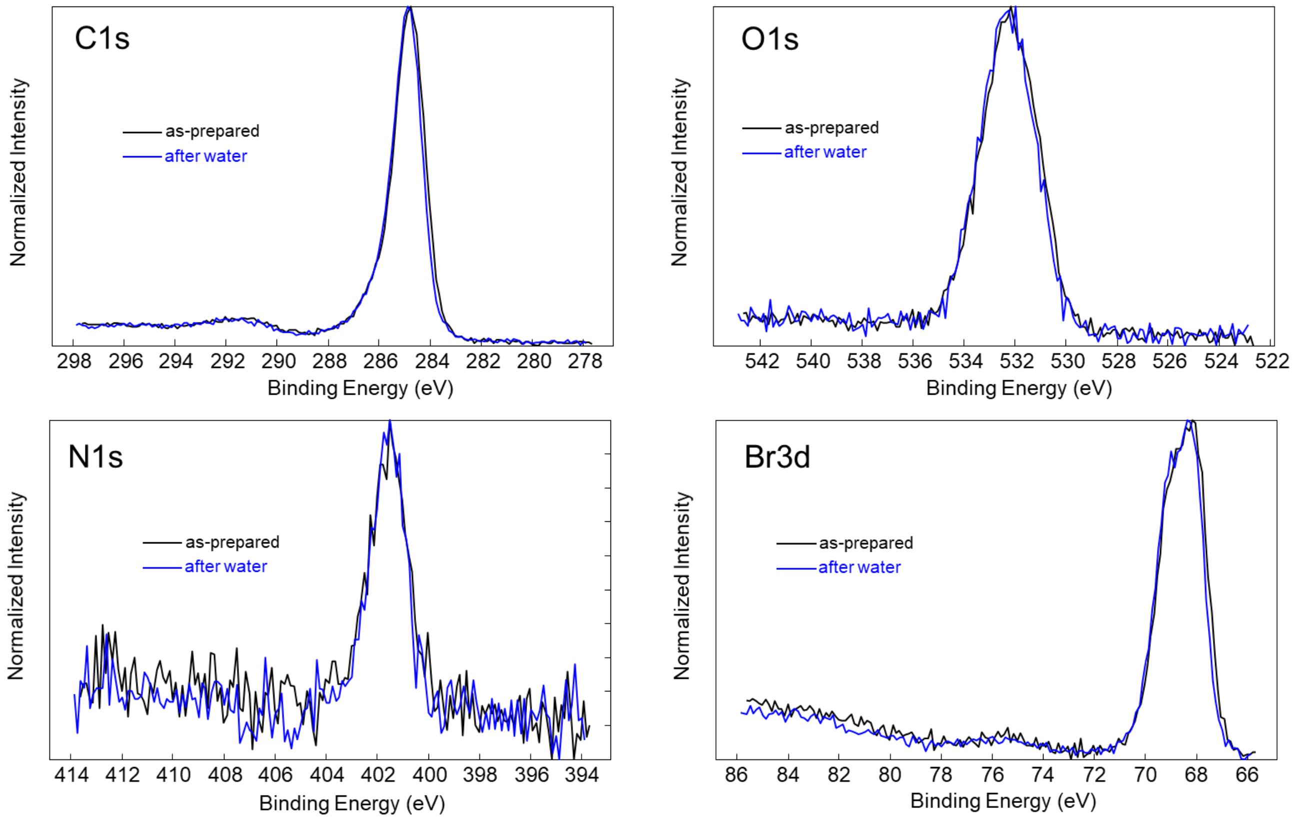

Figure S2. Photoelectron spectroscopy $PhBz_2GeBr_4$. Related to Figure 2. XPS spectra of as-prepared $PhBz_2GeBr_4$ (black), and after 24 h water treatment (blue).

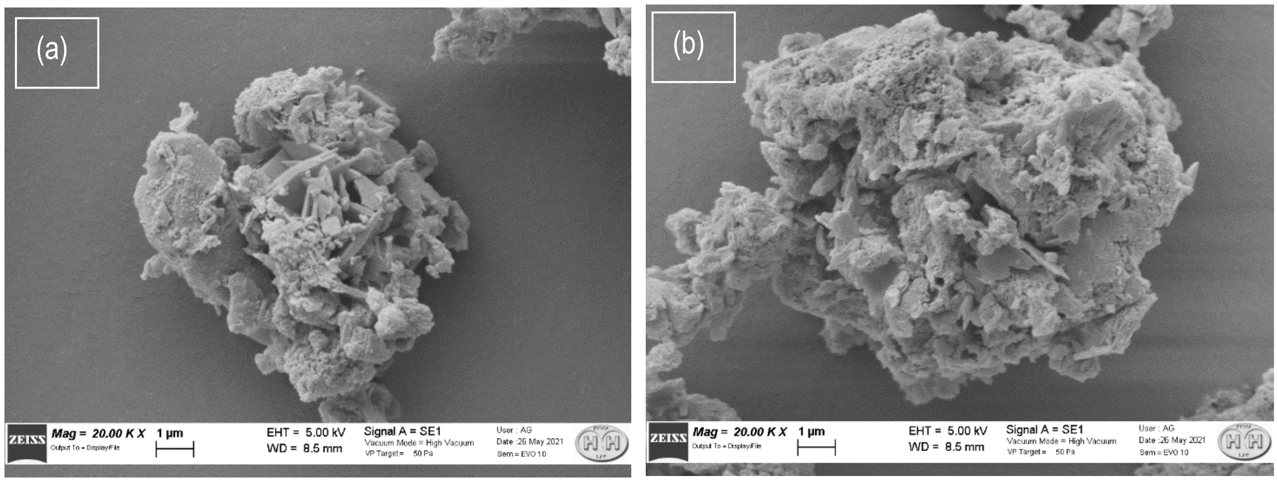

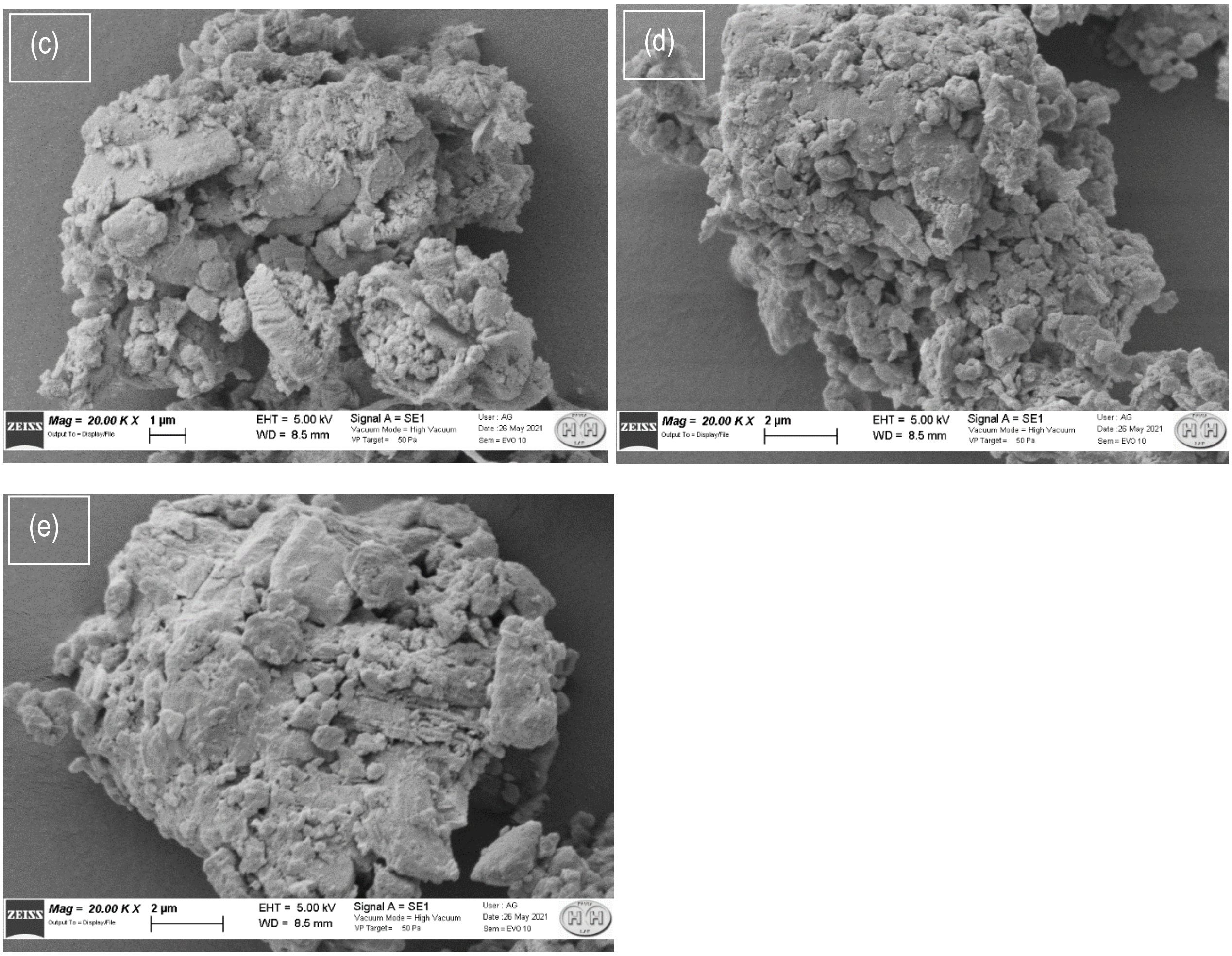

Figure S3. Scanning Electron Microscopy. SEM images of the $PhBz_2GeBr_4$/g-C_3N_4 composites at 1 (a), 2.5 (b), 2.5 (c), 5 (d), and 15 wt% of MHP.

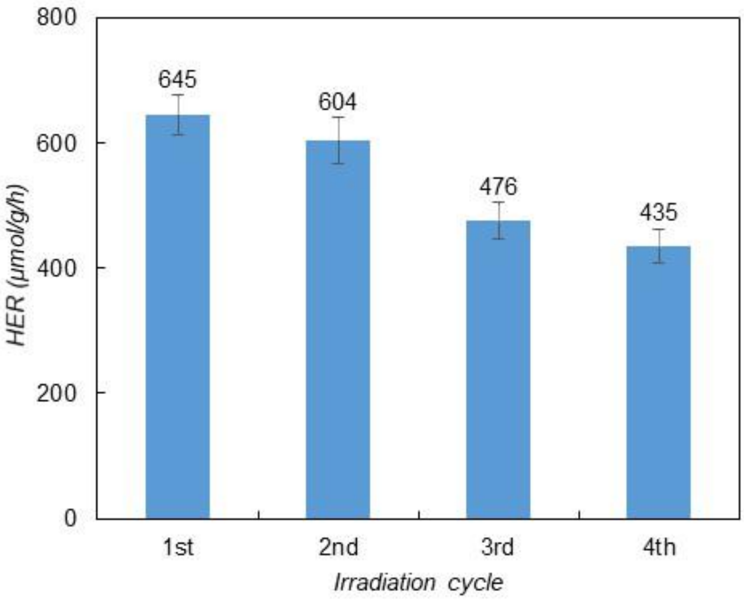

Figure S4. Cyclability test. Related to Figure 4. HERs after four successive photocatalytic cycles (6 h each) for $PhBz_2GeBr_4$ 2.5wt%/g-C_3N_4 (1 g L^{-1} catalyst, 3 wt% Pt, 10% v/v TEOA, simulated solar light (Xenon lamp, 500 W m^{-2}, 300-800 nm, IR-treated soda lime glass UV outdoor filter); RSD < 10% (n=3).

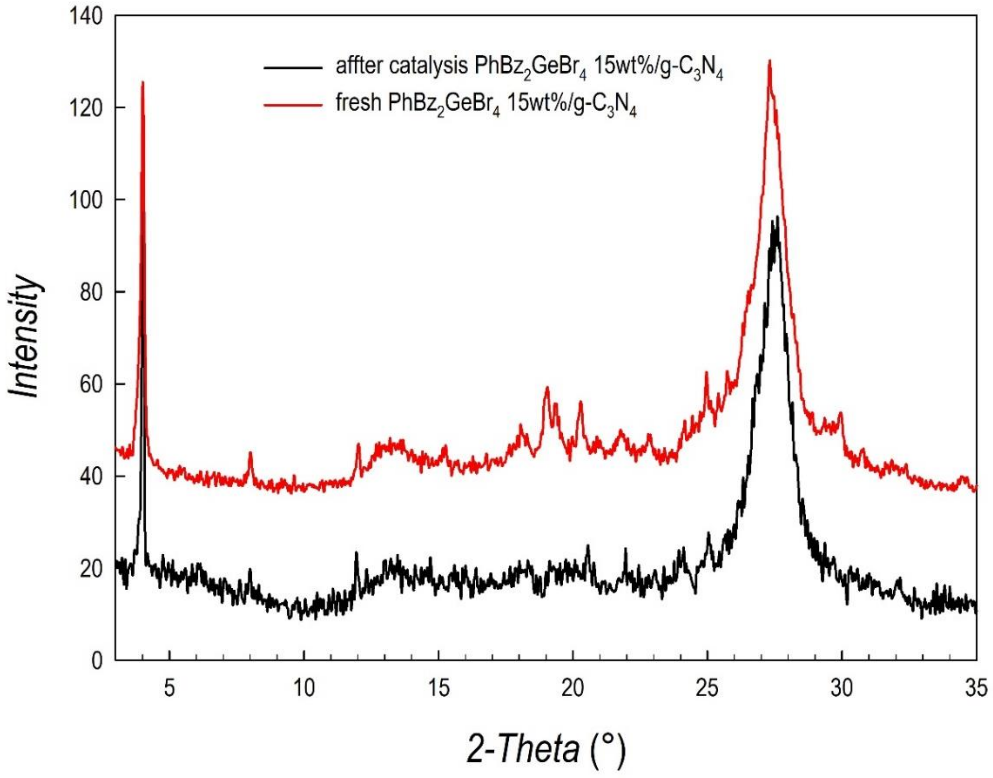

Figure S5. Post-catalysis Diffraction. Related to Figure 4. XRD pattern of 15 wt% $PhBz_2GeBr_4$/g-C_3N_4 before (red) and after (black) photocatalysis.

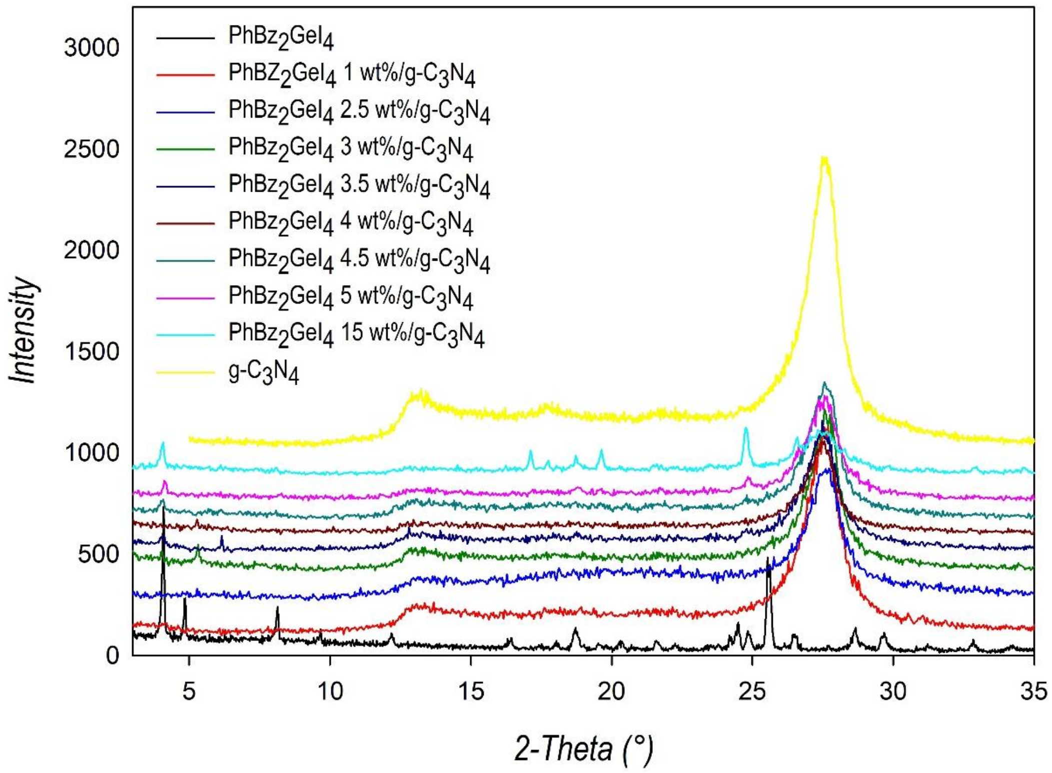

Figure S6. **Diffraction data of $PhBz_2GeI_4$/g-C_3N_4 composites.** Related to Figure 7. XRD patterns of $PhBz_2GeI_4$/g-C_3N_4 composites for different perovskite loadings

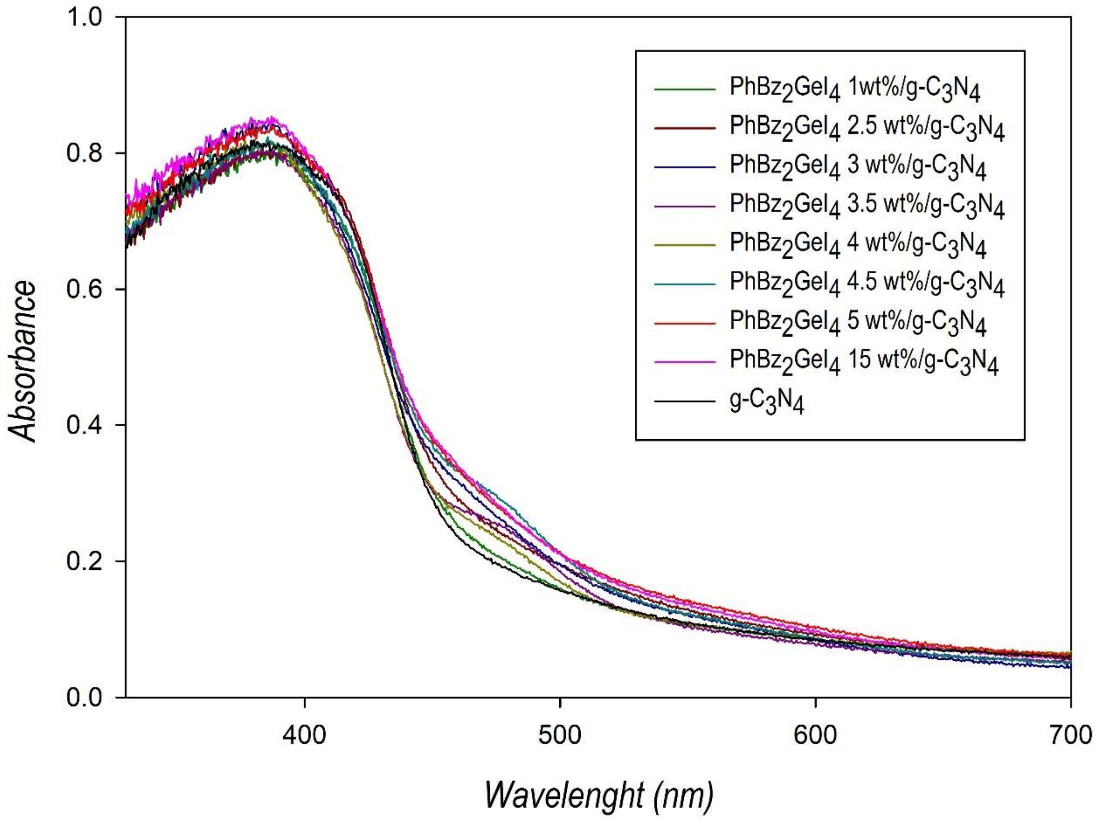

Figure S7. UV-Vis Spectroscopy of $PhBz_2GeI_4$/g-C_3N_4 composites. Related to Figure 7. UV-Vis spectra of $PhBz_2GeI_4$/g-C_3N_4 composites for different perovskite loadings.

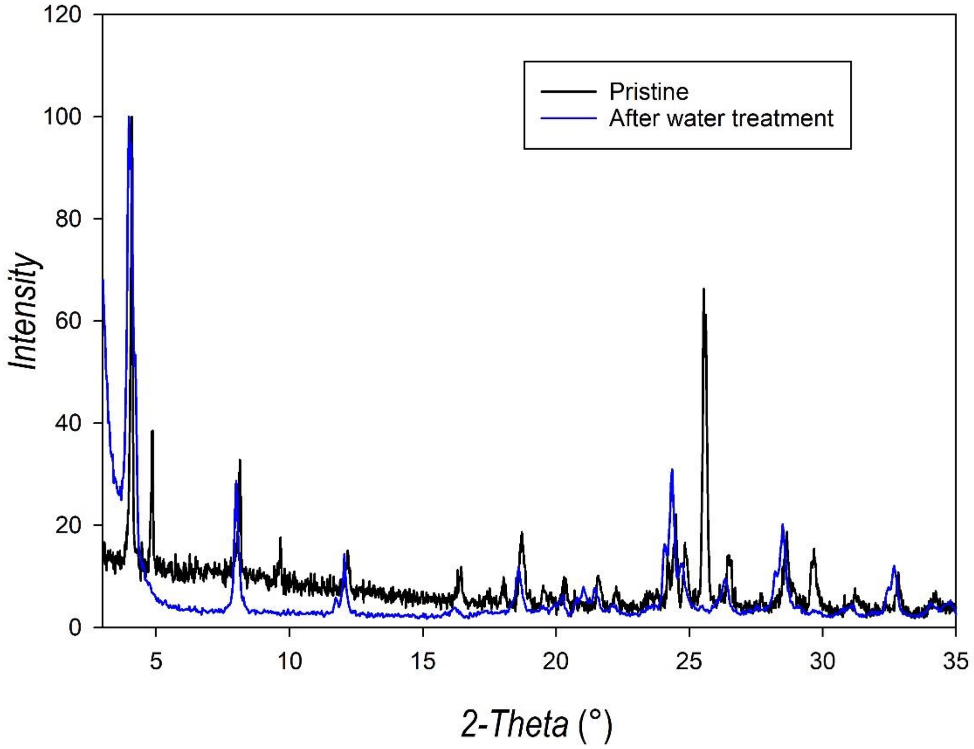

Figure S8. Diffraction after water-treatment. XRD pattern of $PhBz_2GeI_4$ before (black) and after (blue) water treatment.

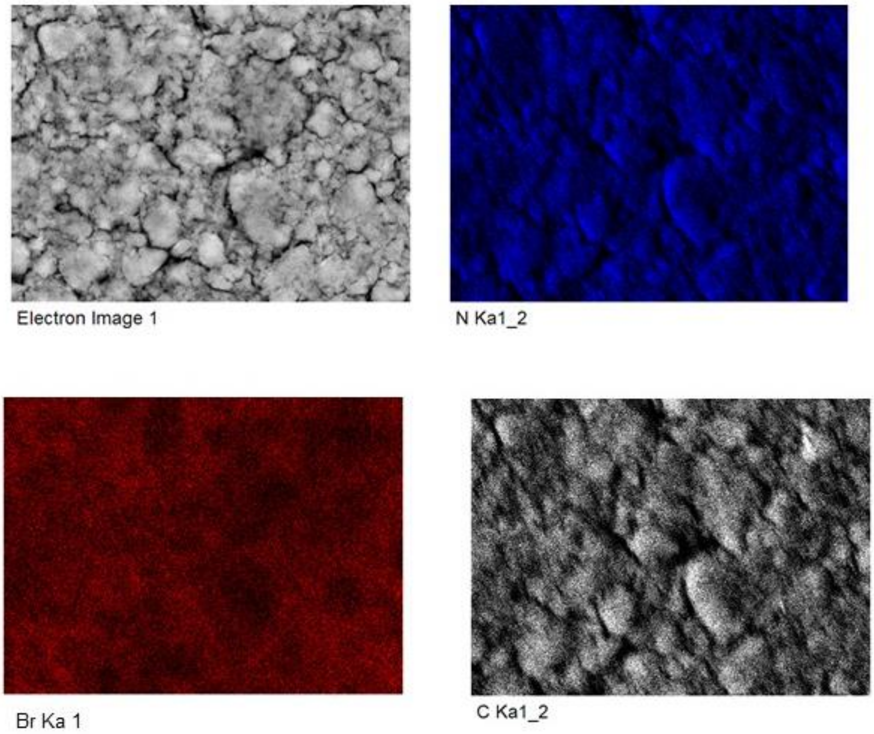

Figure S9. Elemental Mapping. Elemental mapping of $PhBz_2GeBr_4$ 2.5 wt%/g-C_3N_4 composite.

Table S1. Hydrogen Production Results with Biomasses. Related to Figure 4. HERs from model sacrificial biomasses.

	HER (μmol g^{-1} h^{-1})[a]	
	2.5% $PhBZ_2GeBr_4$/g-C_3N_4	3% $PhBz_2GeI_4$/g-C_3N_4
Glucose	55	151
Starch	35	78

[a]RSD < 10% (*n*=3). Conditions: 0.5 g L^{-1} catalyst, 0.5wt% Pt, 0.2 M glucose or 4.5 g L^{-1} starch, simulated solar light (Xenon lamp, 500 W m^{-2}, 300-800 nm, IR-treated soda lime glass UV outdoor filter), 6 h irradiation.

SUPPLEMENTAL NOTES

NOTE S1. Computational Details. Related to Figure 6.

Formation energies

All the calculations have been performed with the CP2K code.[1] Atom-centered Gaussian-type basis functions are used to describe the orbitals. We employ the MOLOPT[2] basis set and use a cutoff of 600 Ha for the plane waves. Core-valence interactions are described by Goedecker-Teter-Hutter pseudopotentials.[3]

To calculate the formation energies from first-principles as reported in Eq. 2 of the main text, we need to build atomistic models (i) of the considered A_2GeX_4 perovskites: $(PEA)_2GeBr_4$, $(BrPEA)_2GEBr_4$, $(BPEA)_2GeI_4$, and $(BPEA)_2GeBr_4$, X(ii) of GeX2 (X=I, Br) and, (iii) of AX. For $(PEA)_2GeBr_4$ and $(BrPEA)_2GEBr_4$, we construct supercells starting from the experimental crystallographic structures (cf. Table S2). Then, we perform density functional theory (DFT) calculations to relax both the coordinates of the atoms and the lattice parameters. These calculations are carried out employing the rVV10 functional, which accounts for van der Waals interactions and has been found to be suited to describe the energetics of layered and 2D materials.[4,5] In fact, the calculated lattice parameters for $(PEA)_2GeBr_4$ and $(BrPEA)_2GEBr_4$ nicely agree with those measured, with differences below 2%. To model $(BPEA)_2GeI_4$, we start from the experimental crystallographic structure of $(BPEA)_2PbI_4$[6] in which we replace Pb atoms with Ge atoms and then we fully relax both coordinates and lattice parameters. Analogously, we obtain an atomistic model of $(BPEA)_2PbBr_4$, by further replacing I with Br.

GeI_2 and $GeBr_2$ are analogously modelled constructing atomistic supercells from the experimentally available crystal structure: a=b=4.13 c=6.75, $\alpha=\beta=90^o$,$\gamma=120^o$ for GeI_2 and a=11.680, b=9.120, c=6.869 $\alpha=\gamma=90^o$, $\beta=101.9^o$ for GeBr2).[7,8] For GeI_2 ($GeBr_2$), we constructed a 4x4x3 (2x2x3) supercell. For AX salts, experimental data were not available for many of them. Therefore, we considered, as a reference, the total energy of an isolated AX dimer for all considered species.

Table S2. **Calculated structural parameters**. Related to Figure 6. Calculated values of the lattice parameters (Å) for the perovskites considered in this study (experimental values from Ref. 9 are given in parenthesis, where available) and number of atoms of the employed supercells.

Perovskite	Lattice parameters (Å)	Bond Angles (º)	Supercell	N. atoms
$(PEA)_2GeBr_4$	(11.406)	80.516	2x2x2	1312
	(11.524)	74.9498		
	(17.441)	90.012		
$(BrPEA)_2GEBr_4$	11.560 (11.396)	90	1x1x2	752
	11.515 (11.596)	90		
	72.810 (71.0480)	90		
$(BPEA)_2GeI_4$	44.565	90	1x2x2	1072
	16.929	90		
	17.360	90		
$(BPEA)_2GeBr_4$	46.911	90	1x2x2	1072
	15.878	90		
	16.623	90		

(100) surfaces of $(BPEA)_2GeX_4$ perovskites

We consider three possible symmetric surface terminations for $(BPEA)_2GeI_4$: (i) stoichiometric (s) (BPEA)I-terminated slab, (ii) non-stoichiometric (ns) (BPEA)I-terminated slab, ns-GeI_2-terminated slab (cf. Fig. S7). The details of the considered slabs are given in Table S3 and the respective structures are shown in Table S3.

For the s-(BPEA)I-terminated slab, the surface energy $\gamma(s)$ is simply given by:

$$\gamma(s) = \frac{E_{\mathrm{slab}}(s) - nE_{\mathrm{bulk}}}{2A}$$

where $E_{\mathrm{slab}}(s)$ is the total energy of the stoichiometric slab, E_{bulk} is the total energy per formula unit of the bulk material, n the number of formula units in the slab supercell, and *A* is the surface area of the slab. For the non-stoichiometric slabs, we notice that these are generated when cleaving the surface along the plane perpendicular to apical Ge-Br bonds. Therefore, we first need to consider the cleavage energy E_{cl} which is defined as:

$$E_{cl} = \frac{1}{4}[E^{\mathrm{v}}_{\mathrm{slab}}(\mathrm{ns1}) + E^{\mathrm{v}}_{\mathrm{slab}}(\mathrm{ns2}) - \ nE_{\mathrm{bulk}}]$$

where $[E^{\mathrm{v}}_{\mathrm{slab}}(\mathrm{ns1})$ and $E^{\mathrm{v}}_{\mathrm{slab}}(\mathrm{ns2})$ are the total energies of the slabs generated upon cleavage (i.e. without relaxation), while n is the number of formula units corresponding to the non-cleaved system. For each non-stoichiometric slab, the surface energy is then defined as:

$$\gamma(ns) = \frac{E_{cl} + E_{rel}}{A}$$

with:

$$E_{rel} = \frac{E_{\mathrm{slab}}(\mathrm{ns}) - E^{\mathrm{v}}_{\mathrm{slab}}(\mathrm{ns})}{2},$$

where $E_{\mathrm{slab}}(\mathrm{ns})$ is the total-energy of the relaxed slab. Results calculated at the rVV10 level of theory are collected in Table S3 and clearly demonstrate that the s-(BPEA)I is the most stable termination. For this reason, we employ this termination also for both $(BPEA)_2GeI_4$ and $(BPEA)_2GeBr_4$ in the calculation of the band alignment.

Table S3. **Calculated structural parameters**. Related to Figure 6. Cell parameters (Å) for the (100) slabs considered in this study (for *z*, the size of the vacuum layer is given in parenthesis), number of atoms of the model, and surface energy γ (eV/Å^2).

Perovskite	Termination	Cell parameters (Å)	γ (eV/Å^2)
$(BPEA)_2GeI_4$	s-(BPEA)I	16.929, 17.360, 100.00 (~30)	0.005
	ns-(BPEA)I	16.929, 17.360, 100.00 (~35)	0.014
	ns-GeI_2	16.929, 17.360, 100.00 (~30)	0.015
$(BPEA)_2GeBr_4$	s-(BPEA)Br	15.878, 16.623, 100.00 (~30)	0.005

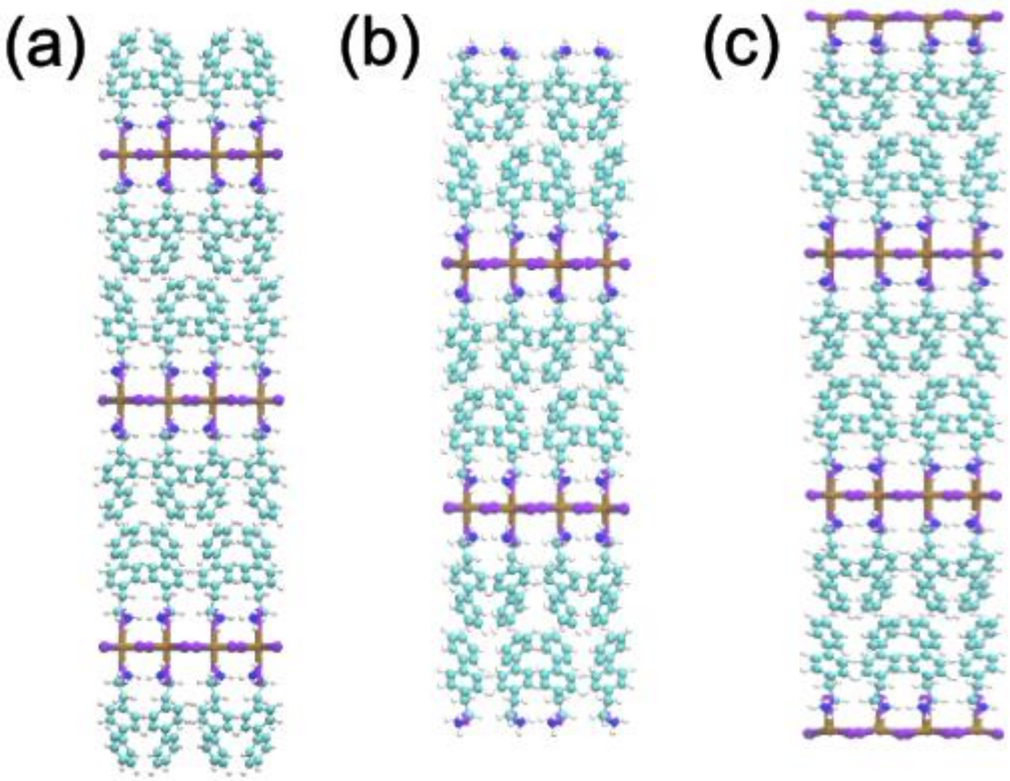

Figure S10. Calculated structural terminations. Related to Figure 6. Stick&ball representation (side view) of the different terminations considered for the (100) surface of $(BPEA)_2GeI_4$: (a) s-(BPEA)I, (b) ns-(BPEA)I, (c) ns-GeI_2. Ge atoms are depicted in ochre, I in violet, C in cyan, N in blue, and H in white.

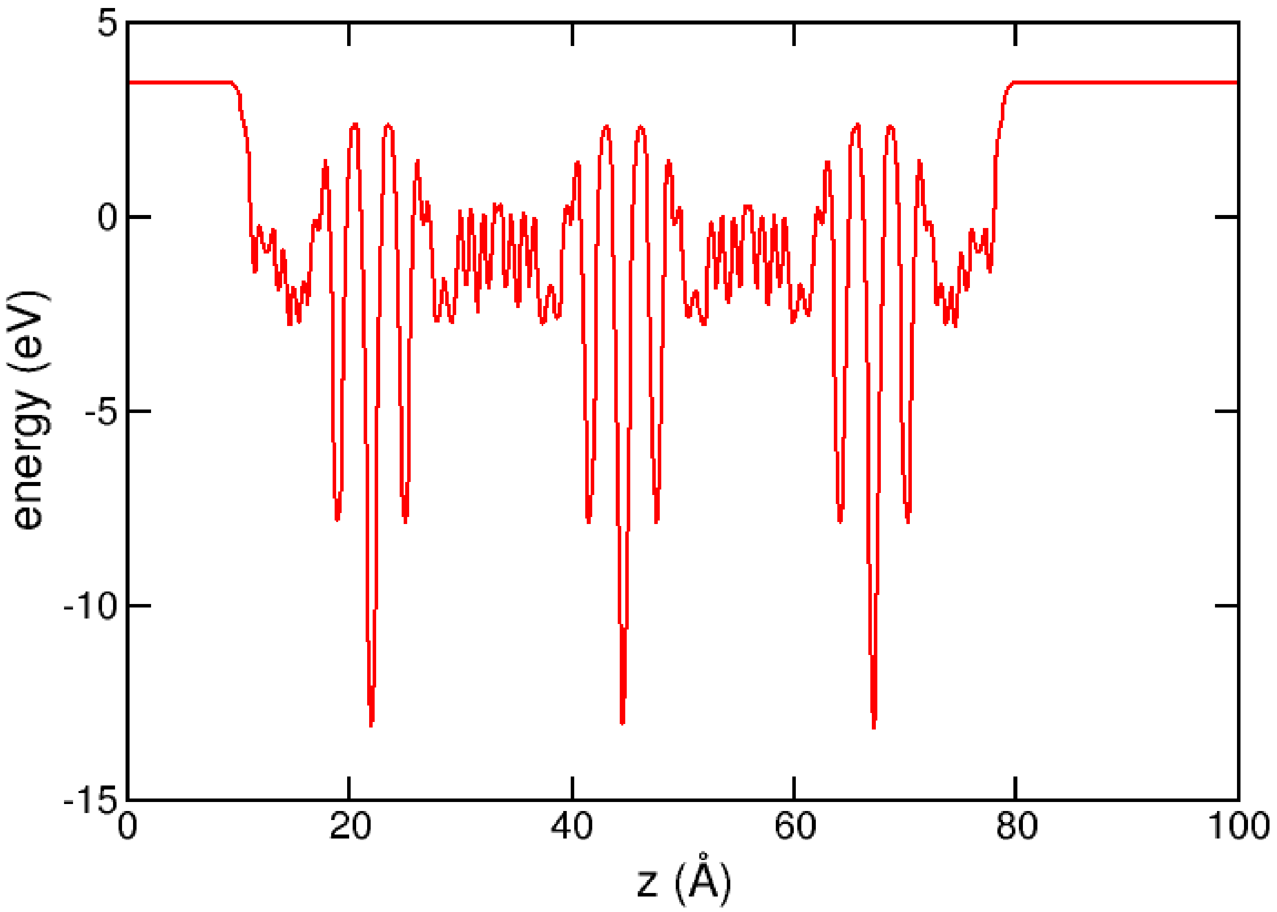

Figure S11. Electrostatic Potential. Related to Figure 6.v Average electrostatic potential referred to the vacuum level across the semiconductor-vacuum interface for $(BPEA)_2GeI_4$.

Band Alignment

We here employ electronic-structure calculations at the hybrid DFT level of theory, in order to align the band edges of the studied Ge-based perovskites with respect to the vacuum level. Hybrid-DFT calculations have been carried out with CP2K using the auxiliary density matrix method to speed up the calculation of exact exchange. To this end, we employ the cFIT auxiliary basis set.[10]

We note that the band alignment for g-C_3N_4 (reported in Fig. 7 of the main text) has been carried out in a previous study.[11] Therefore, we here report on the results achieved for $(BPEA)_2GeI_4$ and $(BPEA)_2GeBr_4$. First, we reproduce the experimental band gap of the perovskites by tuning the the fraction of Fock exchange α of the PBE0 functional [12,13] . This method has been found to produce ionization potentials, electron affinities and energy levels at the semiconductor-water interface in remarkable agreement with the experiment.[14,15] In fact, mean average errors of ~0.2 eV have been estimated for these quantities in screenings performed on a large set of semiconductors.[14]

The band edges are then aligned with respect to the vacuum level, by determining the electrostatic-potential line-ups across the surfaces (cf. Fig. S8). We note that the flat potential across the vacuum region ensures that no residual electrical field is present in the studied slabs (cf. Figure S7). Further, we position the standard hydrogen electrode (SHE) with respect to the vacuum level. In particular, we employ the theoretical alignment

presented in Ref. 13, which has been achieved combining molecular dynamics simulation of a water-vacuum interface with a computational hydrogen electrode.[16,17] The alignment scheme presented in Fig. 7 of the main text is completed including the measured TEOA/TEOA+ redox level.[18]

Solvation Gibbs free energies of A cations

We calculated the solvation Gibbs free energies $\Delta G^0_{\mathrm{solv}}$ of selected A-site cations (cf. Figure S9) in aqueous environment. This quantity is defined as:

$$\Delta G^0_{\mathrm{solv}}(\mathrm{A}^+) = G^0_{\mathrm{solv}}(\mathrm{A}^+) - G^0_{\mathrm{vac}}(\mathrm{A}^+)$$

where $G^0_{\mathrm{solv}}(\mathrm{A}^+)$ and $G^0_{\mathrm{vac}}(\mathrm{A}^+)$ are defined as the Gibbs free energy of the solute in aqueous solution and in vacuum. These quantities are calculated using the Gaussian09 program package.[19] In particular, we employ the B3LYP exchange–correlation functional,[20] with a 6-31++G** basis set for C N H and the ECP lanl2dz pseudopotential for Br and I.[21,22] The implicit effect of the solvent was included performing calculations with the conductor-like polarizable continuum model (C-PCM).[23] Furthermore, to account also for the solvent explicitly, we included three water molecules for each considered cations. Calculated results are reported in Table S4.

Table S4. Solvation Energies. Calculated values of $\Delta G^0_{solv}(A^+)$ (eV) for A-site cations employed in this work and in Ref. 7

**A-site cation**	$\boldsymbol{\Delta G^0_{\mathrm{solv}}(\mathrm{A}^+)}$
$BPEA^+$	-2.61
$PhBz^+$	-2.62
PEA^+	-2.80
$FPEA^+$	-2.84
BrPEA	-2.86
BzA^+	-2.92

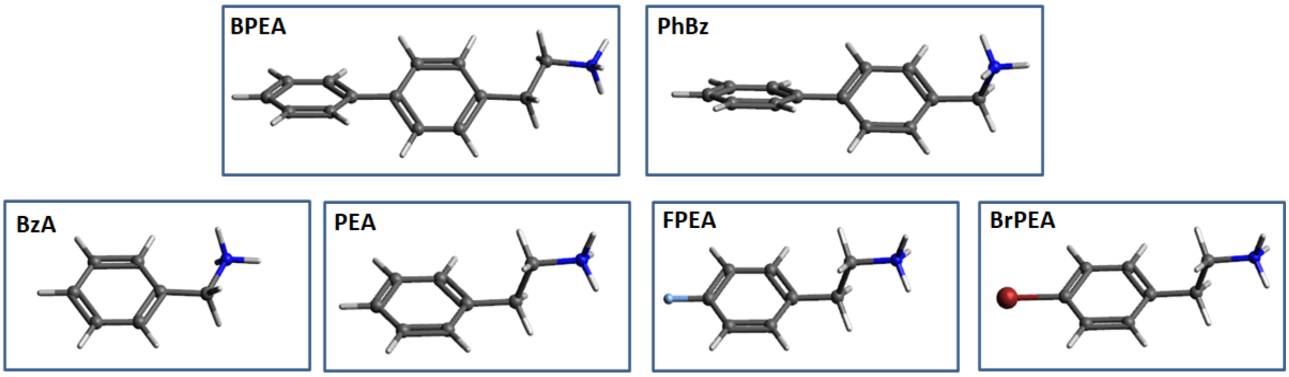

Figure S12. Cation structure representation for solvation calculations. A-site cations considered for the calculation of solvation Gibbs free energy in water. C in grey, N in blue, and H in white.

1. VandeVondele, J., Krack, M., Mohamed, F., Parrinello, M., Chassaing, T., and Hutter, J. (2005). Quickstep: Fast and accurate density functional calculations using a mixed Gaussian and plane waves approach. Comput. Phys. Commun. *167*, 103-128. https://doi.org/10.1016/j.cpc.2004.12.014.
2. VandeVondele, J., and Hutter, J. (2007). Gaussian basis sets for accurate calculations on molecular systems in gas and condensed phases. J. Chem. Phys. *127*, 114105. 10.1063/1.2770708.
3. Goedecker, S., Teter, M., and Hutter, J. (1996). Separable Dual-Space Gaussian Pseudopotentials. Phys. Rev. B *54*, 1703-1710. 10.1103/PhysRevB.54.1703.
4. Vydrov, O.A., and Van Voorhis, T. (2010). Nonlocal van der Waals density functional: The simpler the better. J. Chem. Phys. *133*, 244103. 10.1063/1.3521275.
5. Sabatini, R., Gorni, T., and de Gironcoli, S. (2013). Nonlocal van der Waals density functional made simple and efficient. Phys. Rev. B *87*, 041108. 10.1103/PhysRevB.87.041108.
6. Venkatesan, N.R., Mahdi, A., Barraza, B., Wu, G., Chabinyc, M.L., and Seshadri, R. (2019). Enhanced yield-mobility products in hybrid halide Ruddlesden–Popper compounds with aromatic ammonium spacers. Dalton Trans. *48*, 14019-14026. 10.1039/C9DT03074C.
7. Rouse, R.C., Peacor, D.R., and Maxim, B.R. (1977). The crystal structure of germanium dibromide. Z. Kristallogr. Krist. *145*, 161-171.
8. Powell, H.M., and Brewer, F.M. (1938). 35. The structure of germanous iodide. J. Am. Chem. Soc., 197-198.
9. Chiara, R., Morana, M., Boiocchi, M., Coduri, M., Striccoli, M., Fracassi, F., Listorti, A., Mahata, A., Quadrelli, P., Gaboardi, M., et al. (2021). Role of spacer cations and structural distortion in two-dimensional germanium halide perovskites. J. Mater. Chem. C *9*, 9899-9906. 10.1039/D1TC02394B.
10. Guidon, M., Hutter, J., and VandeVondele, J. (2010). Auxiliary Density Matrix Methods for Hartree−Fock Exchange Calculations. J. Chem. Theory Comput. *6*, 2348-2364. 10.1021/ct1002225.
11. Romani, L., Speltini, A., Ambrosio, F., Mosconi, E., Profumo, A., Marelli, M., Margadonna, S., Milella, A., Fracassi, F., Listorti, A., et al. (2021). Water-Stable DMASnBr3 Lead-Free Perovskite for Effective Solar-Driven Photocatalysis. Angew. Chem. Int. 60, 7, 3611-3618. https://doi.org/10.1002/anie.202007584.
12. Perdew, J.P., Ernzerhof, M., and Burke, K. (1996). Rationale for mixing exact exchange with density functional approximations. J. Chem. Phys. *105*, 9982-9985. 10.1063/1.472933.
13. Adamo, C., and Barone, V. (1999). Toward reliable density functional methods without adjustable parameters: The PBE0 model. J. Chem. Phys. *110*, 6158-6170. 10.1063/1.478522.
14. Guo, Z., Ambrosio, F., Chen, W., Gono, P., and Pasquarello, A. (2018). Alignment of Redox Levels at Semiconductor–Water Interfaces. Chem. Mater. *30*, 94-111. 10.1021/acs.chemmater.7b02619.
15. Ambrosio, F., Wiktor, J., and Pasquarello, A. (2018). pH-Dependent Catalytic Reaction Pathway for Water Splitting at the BiVO4–Water Interface from the Band Alignment. ACS Energy Lett. *3*, 829-834. 10.1021/acsenergylett.8b00104.
16. Ambrosio, F., Guo, Z., and Pasquarello, A. (2018). Absolute Energy Levels of Liquid Water. J. Phys. Chem. Lett. *9*, 3212-3216. 10.1021/acs.jpclett.8b00891.
17. Ambrosio, F., Miceli, G., and Pasquarello, A. (2015). Redox levels in aqueous solution: Effect of van der Waals interactions and hybrid functionals. J. Chem. Phys. *143*, 244508. 10.1063/1.4938189.
18. Sampaio, R.N., Grills, D.C., Polyansky, D.E., Szalda, D.J., and Fujita, E. (2020). Unexpected Roles of Triethanolamine in the Photochemical Reduction of CO2 to Formate by Ruthenium Complexes. J. Am. Chem. Soc. *142*, 2413-2428. 10.1021/jacs.9b11897.
19. Frisch, M.J. et al. (2009). Gaussian 09 . Gaussian, Inc., Wallingford CT, 2016.
20. Becke, A.D. (1993). Density-Functional Thermochemistry. III. The Role of Exact Exchange. J. Chem. Phys. *98*, 5648-5652. 10.1063/1.464913.
21. Ditchfield, R., Hehre, W.J., and Pople, J.A. (1971). Self-Consistent Molecular-Orbital Methods. IX. An Extended Gaussian-Type Basis for Molecular-Orbital Studies of Organic Molecules. J. Chem. Phys. *54*, 724-728. 10.1063/1.1674902.
22. Hay, P.J., and Wadt, W.R. (1985). Ab initio effective core potentials for molecular calculations. Potentials for K to Au including the outermost core orbitals. J. Chem. Phys. *82*, 299-310. 10.1063/1.448975.
23. Cossi, M., Rega, N., Scalmani, G., and Barone, V. (2003). Energies, structures, and electronic properties of molecules in solution with the C-PCM solvation model. J. Comput. Chem. *24*, 669-681. https://doi.org/10.1002/jcc.10189.
