# Supplementary material for: Air- and water-stable and photocatalytically active germanium-based 2D perovskites by organic spacer engineering
Source: Cell Rep Phys Sci. 2023 Jan 18;4(1):101214. doi: 10.1016/j.xcrp.2022.101214 (PMC10246422; doi:10.1016/j.xcrp.2022.101214)
Supplement: Document S2. Article plus supplemental information [file mmc2.pdf]

Cell Reports
Physical Science

CellPress
OPEN ACCESS

Article

Air- and water-stable and photocatalytically active germanium-based 2D perovskites by organic spacer engineering

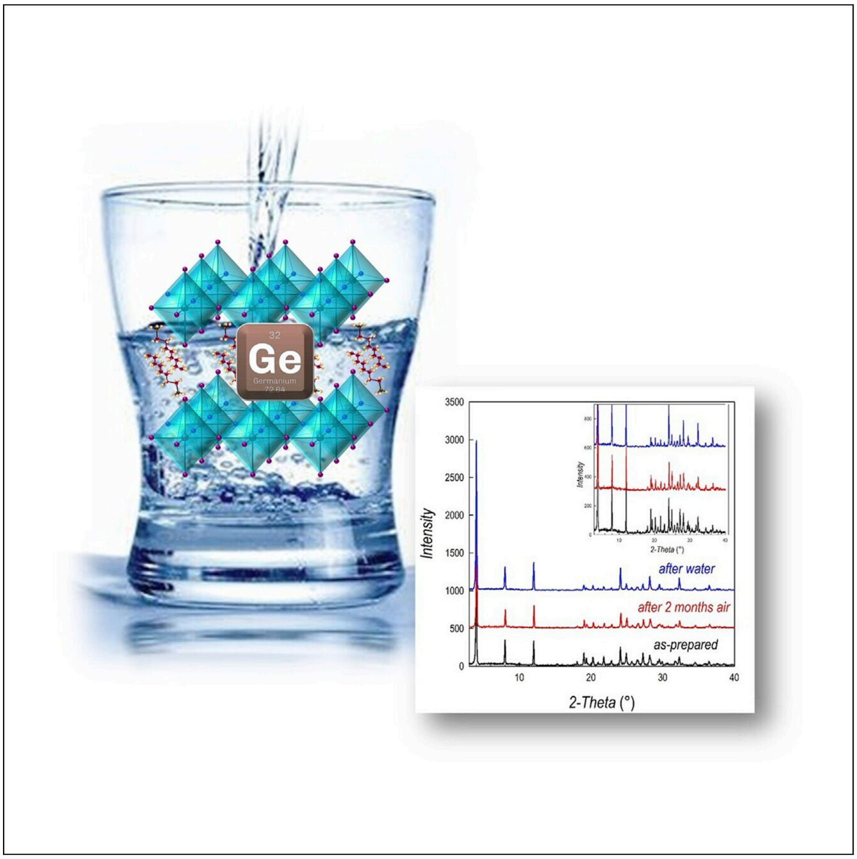

Metal halide perovskite photocatalysis is widely studied in several applied fields. In this work, Romani et al. demonstrate 2D Ge-containing perovskite engineering to improve air and water resistance. Such characteristics are then exploited in solar-driven hydrogen generation in an aqueous environment.

Lidia Romani, Andrea Speltini, Rossella Chiara, ..., Maria Medina-Llamas, Filippo De Angelis, Lorenzo Malavasi

andrea.listorti@uniba.it (A.L.)
francesco.ambrosio@unibas.it (F.A.)
lorenzo.malavasi@unipv.it (L.M.)

Highlights

Preparation of 2D Ge-containing metal halide perovskite

Demonstration of a strategy to induce air/water resistance by cation engineering

Strategy to create heterojunctions between Ge-perovskite and carbon nitride

Demonstration of active photocatalytic activity in hydrogen generation

Romani et al., Cell Reports Physical Science 4, 101214
January 18, 2023 © 2022 The Author(s).
https://doi.org/10.1016/j.xcrp.2022.101214

Cell Reports
Physical Science

CellPress
OPEN ACCESS

Article

Air- and water-stable and photocatalytically active germanium-based 2D perovskites by organic spacer engineering

Lidia Romani,[1,2] Andrea Speltini,[1] Rossella Chiara,[1] Marta Morana,[1] Clarissa Coccia,[1] Costanza Tedesco,[1] Vincenza Armenise,[3] Silvia Colella,[4] Antonella Milella,[3] Andrea Listorti,[3,*] Antonella Profumo,[1] Francesco Ambrosio,[5,6,7,*] Edoardo Mosconi,[7] Riccardo Pau,[8,9] Federico Pitzalis,[8] Angelica Simbula,[8] Damiano Ricciarelli,[10] Michele Saba,[8] Maria Medina-Llamas,[1,10] Filippo De Angelis,[7,11,12] and Lorenzo Malavasi[1,13,*]

SUMMARY

There is increasing interest in the role of metal halide perovskites for heterogeneous catalysis. Here, we report a Ge-based 2D perovskite material that shows intrinsic water stability realized through organic cation engineering. Incorporating 4-phenylbenzilammonium (PhBz) we demonstrate, by means of extended experimental and computational results, that $PhBz_2GeBr_4$ and $PhBz_2GeI_4$ can achieve relevant air and water stability. The creation of composites embedding graphitic carbon nitride ($g\text{-}C_3N_4$) allows a proof of concept for light-induced hydrogen evolution in an aqueous environment by 2D Ge-based perovskites thanks to the effective charge transfer at the heterojunction between the two semiconductors.

INTRODUCTION

Metal halide perovskites (MHPs) are attracting huge interest for their possible application in heterogeneous photocatalysis following the recent synthesis of materials and heterostructures that have been found to be efficient for a plethora of photocatalyzed chemical reactions, including hydrogen generation and CO_2 reduction.[1–17] A major limitation of MHPs in photocatalysis is their limited water stability, which derives from the high ionic character of the metal halide framework.[2,18,19] In this context, 2D-layered MHPs may be particularly suitable to overcome this issue in virtue of the vast range of organic spacers that can be inserted as a protective barrier between the inorganic perovskite layer(s).[20] In fact, upon the introduction of a highly hydrophobic organic spacer in the perovskite structure, significant moisture and water stability could be achieved, and examples exist of systems forming a suspension in water instead of being dissolved.[8,21–23] In this respect, recently, some Bi- and Sn-based perovskites with improved water resistance were found to show significant visible-light photocatalytic activity of hydrogen photogeneration and organic dye degradation, which was further enhanced by designing heterostructures with graphitic carbon nitride ($g\text{-}C_3N_4$).[8,21] Similar strategies have been used also by other authors on the $Cs_3Bi_2I_9$ perovskite derivative.[7,11]

While materials engineering has been successful for the synthesis of lead-free MHP photocatalysts, there are no reports about any photoactive Ge halide perovskite. Recently, a series of Ruddlesden-Popper (RP) 2D Ge bromide perovskites (n = 1), namely A_2GeBr_4, with A = $C_6H_4CH_2CH_2NH_3$ (phenylethylammonium [PEA]); $BrC_6H_4CH_2CH_2$

[1]Department of Chemistry and INSTM, University of Pavia, Via Taramelli 16, 27100 Pavia, Italy
[2]Tecnologie di Generazione e Materiali, Ricerca sul Sistema Energetico - RSE S.p.A., Via Rubattino 54, 20134 Milano, Italy
[3]Department of Chemistry, University of Bari "Aldo Moro," via Orabona 4, 70126 Bari, Italy
[4]National Research Council, Institute of Nanotechnology (CNR-NANOTEC), c/o Department of Chemistry, University of Bari "Aldo Moro," via Orabona 4, 70126 Bari, Italy
[5]Dipartimento di Scienze, University of Basilicata, Viale dell'Ateno Lucano, 10, 85100 Potenza, Italy
[6]Department of Chemistry and Biology "A. Zambelli," University of Salerno, Via Giovanni Paolo II 132, 84084 Fisciano, Salerno, Italy
[7]Computational Laboratory for Hybrid/Organic Photovoltaics (CLHYO), Istituto CNR di Scienze e Tecnologie Chimiche "Giulio Natta" (CNR-SCITEC), via Elce di Sotto 8, 06123 Perugia, Italy
[8]Department of Physics, University of Cagliari, Cittadella Universitaria S.P. Monserrato-Sestu km 0.7, 09042 Monserrato, Italy
[9]Zernike Institute for Advanced Materials, University of Groningen, Nijenborgh 4, 09747 Groningen, the Netherlands
[10]Unidad Académica Preparatoria, Plantel II, Universidad Autónoma de Zacatecas, Zacatecas, Zacatecas 98068, México
[11]Department of Chemistry, Biology and Biotechnology, University of Perugia, via Elce di Sotto 8, 06123 Perugia, Italy
[12]Department of Natural Sciences & Mathematics, College of Sciences & Human Studies, Prince Mohammad Bin Fahd University, Dhahran 34754, Saudi Arabia
[13]Lead contact
*Correspondence: andrea.listorti@uniba.it (A.L.), francesco.ambrosio@unibas.it (F.A.), lorenzo.malavasi@unipv.it (L.M.)
https://doi.org/10.1016/j.xcrp.2022.101214

© 2022 The Author(s).
This is an open access article under the CC BY license (http://creativecommons.org/licenses/by/4.0/).

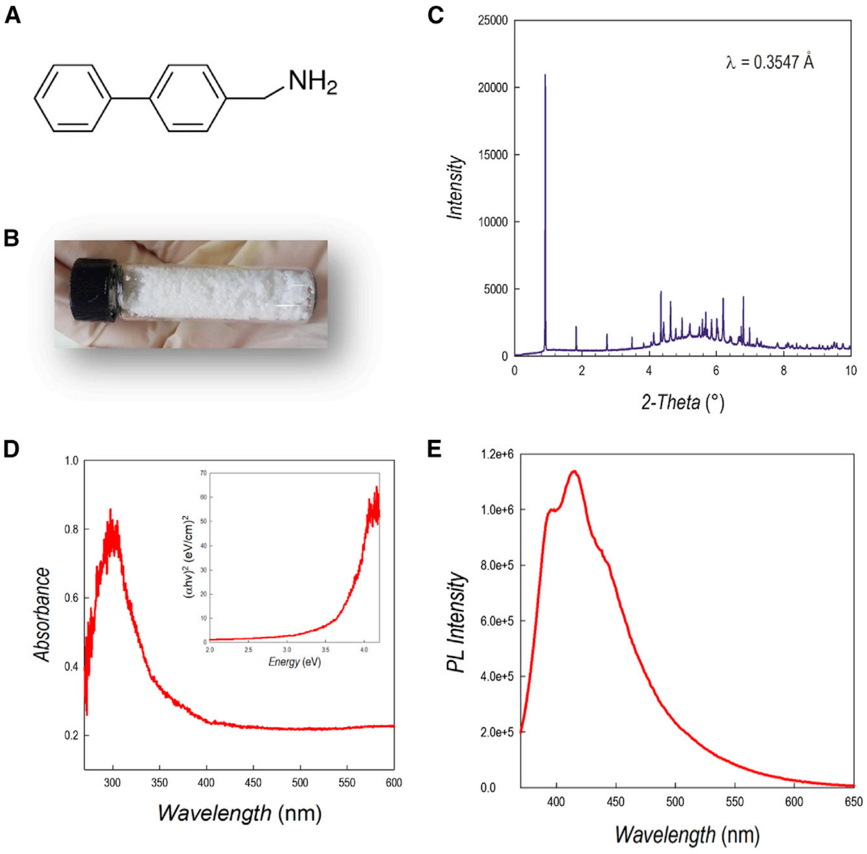

Figure 1. Structural and optical properties of $PhBz_2GeBr_4$

(A) Sketch of the chemical formula of 4-phenylbenzilamine.

(B) Picture showing the appearance of the powdered sample.

(C) SR-XRD pattern of $PhBz_2GeBr_4$ collected at 0.3547 Å.

(D) Absorption spectra of $PhBz_2GeBr_4$ (inset: Tauc plot).

(E) PL spectra of $PhBz_2GeBr_4$.

NH_3 (Br-phenylethylammonium [BrPEA]); $FC_6H_4CH_2CH_2NH_3$ (F-phenylethylammonium [FPEA]); and $C_6H_4CH_2NH_3$ (benzylammonium [BzA]), showed air stability but not water tolerance.[24] Therefore, we further extended this quest by designing a 2D composition including the 4-phenylbenzilammonium (PhBz) spacer cation (Figure 1A). This cation presents extended π-conjugated systems, creating a unique condition in which strong intra-layer van der Waals (vdW) interactions are established, substantially stabilizing the resulting bulk perovskite. Thanks to this stabilization, we demonstrate improved air and water stability in 2D Ge perovskites containing the PhBz cation. Such stability is exploited in the construction of heterojunctions with g-C_3N_4 and their application in the solar-driven hydrogen production in aqueous environment.

RESULTS AND DISCUSSION

$PhBz_2GeBr_4$ perovskite structure and stability

The novel $PhBz_2GeBr_4$ perovskite was synthesized by means of wet-chemistry route as described in the experimental procedures. The powder has a white color (Figure 1B), and the corresponding high-resolution synchrotron radiation (SR) powder X-ray diffraction (XRD) pattern collected at 0.3547 Å is shown in Figure 1C. The indexing of the SR-XRD data (Figure S1A) provided an orthorhombic cell (space group $Pmc2_1$) with refined lattice parameters of a = 6.0260(1), b = 9.1744(2), and

CellPress
OPEN ACCESS

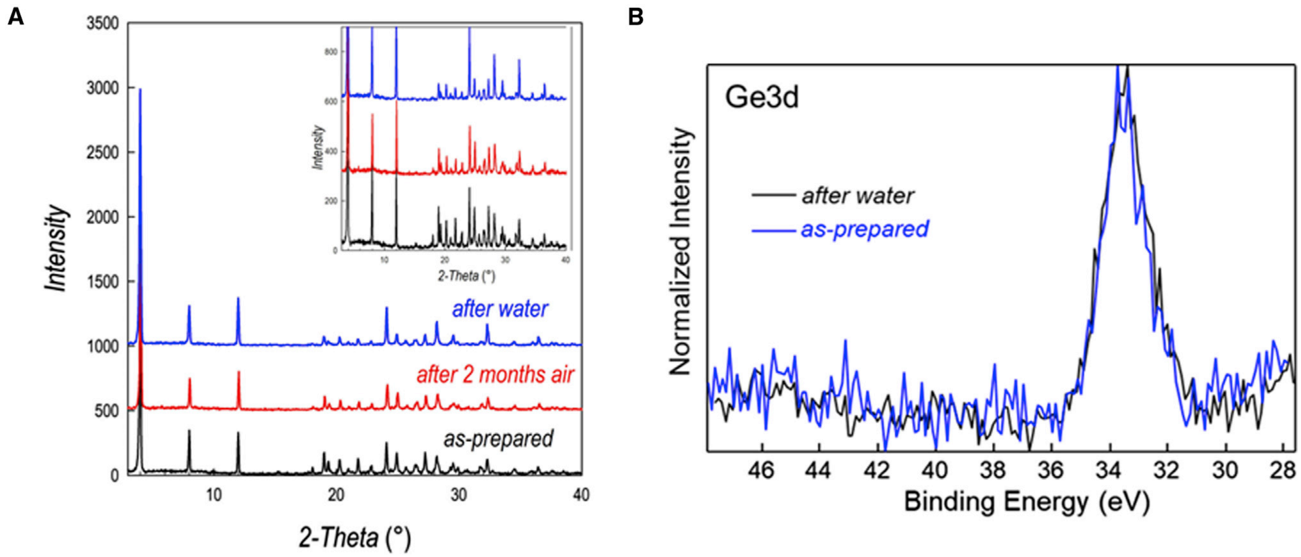

Figure 2. Air and water stability of $PhBz_2GeBr_4$

(A) XRD pattern of as-prepared $PhBz_2GeBr_4$ (black) after 2 months of air exposure (red) and after 24 h water treatment (blue). Inset: focus on a reduced 2-theta range.

(B) Ge3d XPS spectrum of as-prepared $PhBz_2GeBr_4$ (black) and after 24 h water treatment (blue).

c = 44.376(1) Å. There are no literature reports on 2D perovskites containing PhBz cation, therefore a direct comparison with analogous compositions is not possible. The most similar material reported, in terms of organic cation, is $BPEA_2PbI_4$ (BPEA = 2-(4-biphenyl)ethylamine), in which the organic cation has an ethyl linked to the amine group.[25] Even though the central metal and the halide are different with respect to $PhBz_2GeBr_4$, the agreement with the symmetry of the $BPEA_2PbI_4$ crystal suggests a similar organic spacer arrangement as shown in Figure S1B, where $PhBz_2GeBr_4$ diffraction is plotted against the expected Bragg peaks from $BPEA_2PbI_4$. The long c axis is, as well, in a similar range as the one reported here.[25]

The optical properties of $PhBz_2GeBr_4$ have been determined by UV-visible (UV-vis) absorption spectroscopy and photoluminescence (PL) (see Figures 1D and 1E). From the Tauc plot, we estimate a band gap of 3.64 eV, while the PL spectrum shows a structured intense band composed of three contributions with the main emission peak centered at about 415 nm.

To test the stability versus moisture of the synthesized material, as-prepared $PhBz_2GeBr_4$ (kept under argon in glovebox) was exposed to laboratory air ($T \approx 22°C$, relative humidity [RH] $\approx$ 35%) for 2 months. The XRD pattern collected after this time interval shows a perfect match with the pattern of the as-prepared $PhBz_2GeBr_4$ (cf. red and black lines in Figure 2A). Furthermore, $PhBz_2GeBr_4$ powders were dispersed in water and kept under stirring for 24 h, and the diffraction pattern of the powders was found in very good agreement with the as-prepared material (cf. blue versus black patterns in Figure 2A).[9,21] In the rescaled inset of Figure 2A, we highlighted the peaks around 10°–40° to put in prominence the very good agreement between the patterns in the whole diffraction range.

Further evidence of water stability has been assessed through a leaching test. In this assay, $PhBz_2GeBr_4$ has been placed in water under magnetic stirring, and the amount of Ge in the solution has been determined by inductively coupled plasma

optical emission spectroscopy (ICP-OES) (see details in the supplemental information). After 4, 8, 16, and 24 h of stirring, the Ge amounts detected in the solution were 4.3%, 5.7%, 5.9%, and 6%, respectively, of the total amount of Ge present in the perovskites, thus confirming the high water stability and insolubility of $PhBz_2GeBr_4$.

In addition to the clear evidence of air and water structural stability reported above, we applied X-ray photoelectron spectroscopy (XPS) on the as-prepared sample and on the sample after immersion in water. The whole set of recorded spectra is shown in Figure S2, while in Figure 2B, we report the Ge 3d spectrum. The Ge 3d spectra of the two samples reported in Figure 2B are superimposable (as for all the other elements probed, cf. supplemental information), indicating that the Ge ions in immersed samples retain the same oxidation state of as-prepared materials.

The relative position of the Ge 3d peak suggests the possible presence of Ge(IV) on the surface of the material, possibly in the form of a native oxide, which could act as protective layer as recently proposed to justify an enhanced air stability of 2D and 3D Ge perovskites.[26,27] More specifically, the binding energy position of the Ge 3d peaks at 33.4 eV is indicative of a Ge(IV) state and an additional indication of the Ge(IV) oxide formation at the surface of the material derives from the curve-fit analysis of the corresponding O1s spectra (Figure S2), revealing the presence of a component at 531.8 eV typical of GeO2.[28,29]

However, since previously synthesized Ge-based 2D perovskites did not show any water stability, the reason of the improved water stability of $PhBz_2GeBr_4$ may predominantly lay in the bulk properties originating by the significant steric hindrance and hydrophobicity of the organic spacer.[24] Similar water stability has been also observed in some Sn-based MHPs, namely $DMASnBr_3$ and PEA_2SnBr_4.[8,9] In the first material, the mechanism leading to improved stability and effective photocatalytic activity results from the valence band energy stabilization, which should reduce the exposure of the material to oxidants, restoring a value similar to $MAPbI_3$, healing the material from degradation into Sn^{4+} phases and self-p-doping effects.[30,31] On the other hand, the improved stability in PEA_2SnBr_4 was again related to the nature of surface-terminating groups and the presence of hydrophobic PEA moiety.[8] For lead-based perovskites, the highly ionic nature of 3D systems has hampered their use in aqueous environments. For $MAPbI_3$, for example, any photocatalytic activity has been only reported in hydrohalic acids by exploiting the dynamic equilibrium of the dissolution and precipitation of the perovskite in saturated aqueous solutions.[17] More water-stable Pb-containing perovskites have been obtained by exploring the use on long aliphatic chains in 2D systems such as for $(HDA)_2PbI_4$ (HAD = hexadecylammonium).[23] This phase was also used in photocatalytic activity for photoredox C−C bond cleavage and dehydrogenation catalysis but not for hydrogen photogeneration.[23]

$PhBz_2GeBr_4$ hydrogen generation experiments

We exploited the advantage of $PhBz_2GeBr_4$ superior water stability by testing its possible application in photocatalysis. Hydrogen photogeneration characteristics have been determined according to a commonly employed protocol we already applied for other MHPs.[8,9,21] First, we determined the hydrogen evolution rate (HER) of pure $PhBz_2GeBr_4$ under simulated solar light, which turned out to be ~6 μmol g^{-1} h^{-1}, a low but relevant value for a pure MHPs, also considering the relatively high band gap of the materials, 3.6 eV, which corresponds to efficient

CellPress
OPEN ACCESS

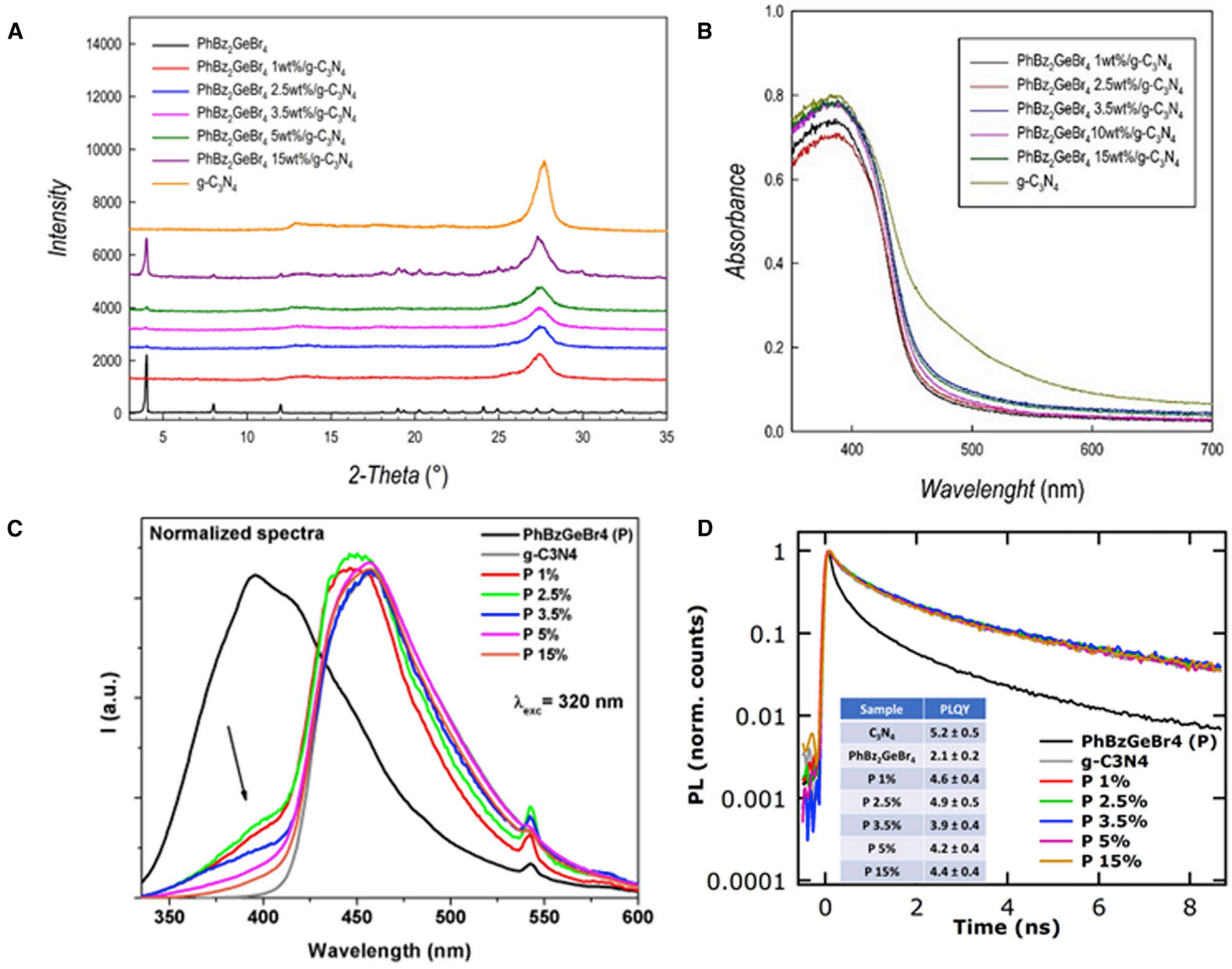

Figure 3. Structural and optical properties of $PhBz_2GeBr_4$/g-C_3N_4 composites

(A) XRD patterns of $PhBz_2GeBr_4$/g-C_3N_4 composites for different perovskite loadings.

(B) UV-vis spectra of $PhBz_2GeBr_4$/g-C_3N_4 composites for different perovskite loadings.

(C) Normalized emission spectra and of $PhBz_2GeBr_4$/g-C_3N_4 composites at different percentages of perovskite loading (wt %). g-C_3N_4 refers to pristine material. λ_{exc} = 320 nm.

(D) Normalized PL decays for the same composites (λ_{exc} = 320 nm; λ_{em} = 450 nm). In the inset, PLQY for all samples obtained with continuous wave (CW) excitation at 405nm.

absorption only in the UV region of the light spectrum (in line with the UV-vis absorption spectrum; Figure 1C). To enhance the photoactivity of the perovskite, we prepared composites with a well-known visible-light-absorbing semiconductor, namely g-C_3N_4. Composites have been synthesized by means of wet-chemistry route as reported in the experimental procedures at different weight percentages (% wt) of MHP relative to g-C_3N_4 (1%, 2.5%, 3.5%, 5%, and 15%). Morphologies of pure samples and composites have been assessed by scanning electron microscopy (SEM) and are reported in Figure S3. Figure 3A reports the XRD patterns of the composites showing the main contribution of g-C_3N_4 up to 15 wt %. We point out that the main peak of $PhBz_2GeBr_4$, located around 4°, is already detectable in the sample containing 2.5 wt % of perovskite. UV-vis spectra of the composites (Figure 3B) show a significant contribution to absorbance deriving from the carbon nitride.

The normalized PL spectra of pure compounds and composites are reported in Figure 3C. Both pristine materials show intense luminescence bands, with $PhBz_2GeBr_4$ peaking at 415 nm and g-C_3N_4 at 455 nm. Their bands falling in different spectral regions allow us to monitor the contribution of the single components to the PL features of the composites. Noticeably, the composites, when excited at a wavelength that allows the absorption of both materials, show a spread PL emission overshadowed by g-C_3N_4 contribution. g-C_3N_4 emission is energetically spread spanning from 410 to 560 nm due to various deactivation paths existing within the energy band diagram of the material.[32] The PL quantum yields (PLQYs) as well as the lifetimes (τ) (Figure 3D) are very similar for pristine and for g-C_3N_4-containing composites. The PLQY varies from 3.9% $\pm$ 0.4% to 5.2% $\pm$ 0.5%, and the average τ is 9.0 $\pm$ 0.5 ns for all specimens, further suggesting the central role played by carbon nitride states in the excitation deactivation path, while the PLQY of pure $PhBz_2GeBr_4$ is lower (2.2% $\pm$ 0.2%), compatible with the shorter PL lifetime. These observations nicely fit the band structure of the junction, which is modeled below (*vide infra*). The normalized PL spectra show, however, some differences among the samples. Features of g-C_3N_4 dominate the spectra at high perovskite loadings, suggesting a highly efficient energy transfer from the perovskite moiety to g-C_3N_4. The low-loading composites show instead some minority features attributable to residual perovskite contribution (Figure 3C). For these compositions, namely 1%, 2.5%, and 3.5% loading of perovskite, the energy transfer appears somehow less effective, suggesting a possible defect-filling mechanism at the g-C_3N_4/perovskite interface (see later in the text). An additional difference related to the shape of the composite emission can be found in the region of its peak (around 440–460 nm); here, the g-C_3N_4 and the high-loading composites show a more intense contribution at low energies, peaking their emission at 458 nm; meanwhile, low-loading composites (1% and 2.5%) show the maximum emission at 444 nm. The shape of the emission band in g-C_3N_4 materials can be attributed to the relative population of diverse energy transitions, withstanding a complex deactivation path for the excitation in g-C_3N_4 materials; in particular, the one at low energies are connected to π^*-π transition, while the one at high energies are related to deactivation of the nitrogen atom lone pair.[32,33] In our systems, the diverse shapes suggest how differences in the relative populations of excitation/deactivation processes exist between low- and high-loading composites. The peculiar optical behavior of composites appears to be a distinctive trait of perovskite carbon nitride systems, as already described elsewhere, suggesting that low levels of perovskite doping may act on funneling the charges upon localized states.[34] Those active sites could be fundamental in boosting the photocatalytic performances of low-loading composites.[34] Such effect faded with increased perovskite loadings.

Finally, we investigated the solar-driven catalytic efficiency of the prepared composites in terms of the HER. The measurements have been carried out by employing current protocols applied in the literature for g-C_3N_4-based composites, *i.e.*, in 10% (v/v) aqueous triethanolamine (TEOA), as a typical sacrificial agent and with Pt (3 wt %) as metal co-catalyst.[35] Figure 4A shows the HER as a function of perovskite loading, while Figure 4B shows the kinetics of the hydrogen evolution for the best-performing composite from Figure 4A, namely 2.5 wt % $PhBz_2GeBr_4$/g-C_3N_4.

Noticeably, the composites display a synergic effect, providing a significant improvement of the HER of about 8 times with respect to pure carbon nitride (81 μmol g^{-1} h^{-1}) and of about 100 times with respect to pure $PhBz_2GeBr_4$ (6 μmol g^{-1} h^{-1}). The maximum HER is found at a relatively low MHP loading of 2.5 wt %, while higher perovskite percentages are not beneficial to improve the photoreaction. For the optimal

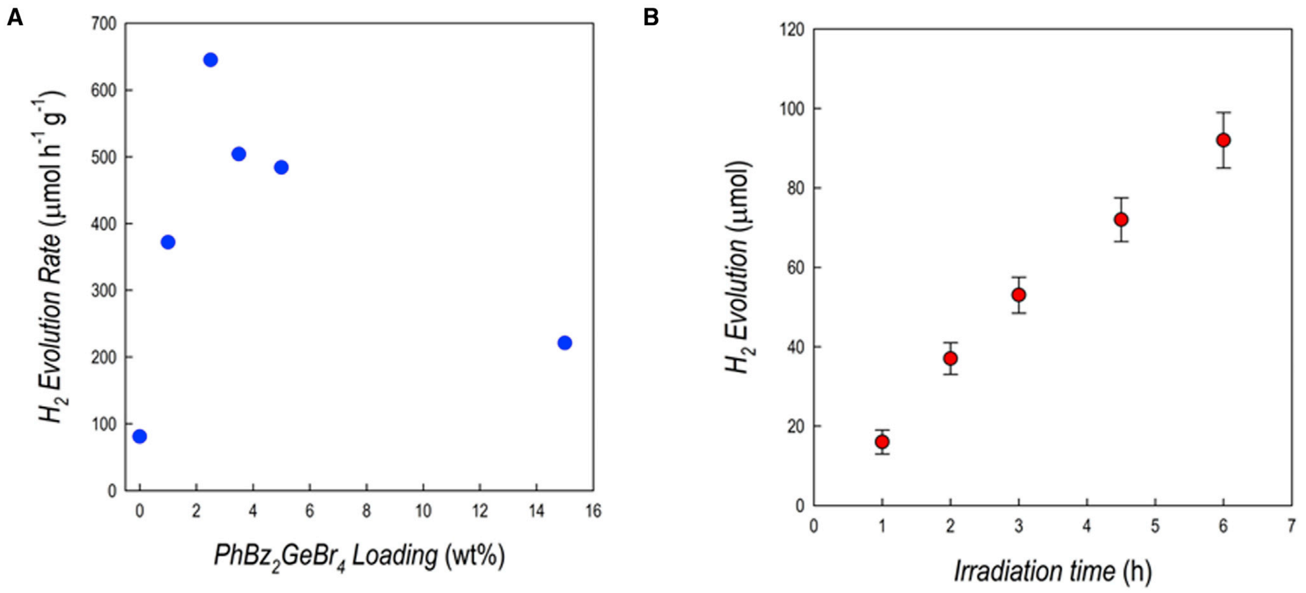

Figure 4. Hydrogen photogeneration performance of $PhBz_2GeBr_4$/g-C_3N_4 composites

(A) Hydrogen evolution rates for $PhBz_2GeBr_4$/g-C_3N_4 composites at different percentages of MHP loading, 6 h irradiation, relative standard deviation (RSD) <10% (n = 3).

(B) Hydrogen evolution profile over irradiation time for the 2.5 wt % $PhBz_2GeBr_4$/g-C_3N_4 composite, RSD <10% (n = 3). Conditions: 1 g L^{-1} catalyst, 10% v/v TEOA, 3 wt % Pt, simulated solar light (Xenon lamp, 500 W m^{-2}, 300–800 nm, IR-treated soda-lime glass UV outdoor filter).

composite, namely at 2.5 wt %, the kinetics of H_2 evolution, reported in Figure 4B, indicates a substantial linear increase of the hydrogen production as a function of time. The value of apparent quantum yield (AQY%), calculated as the percent ratio H_2 moles/incident photons moles, for this composition (2.5 wt % of perovskite) was 5.2%.[36] This is the first evidence of the application of a Ge-based perovskite in solar-driven hydrogen generation; therefore, any comparison with pre-existing literature is not possible. However, the measured rates are similar to those measured for Sn- and Bi-based perovskites.[8,9,34] The composite at 2.5 wt % of $PhBz_2GeBr_4$ has been tested over four successive catalytic cycles by centrifugating, recovering, and subjecting the sample to the same photocatalytic procedure. The HER in the second cycle was about 94% of the initial HER, while in both the third and fourth cycles, it was reduced to about 74% (Figure S4); such a result could be related to the slight, but detectable, Ge leaching reported above. Finally, the catalyst was recovered after a photogeneration test and analyzed by XRD to test the material stability. For the sake of clarity this test was accomplished on the material with 15 wt % loading of $PhBz_2GeBr_4$ since it showed the most evident reflections from the perovskite. The patterns of fresh $PhBz_2GeBr_4$ 15 wt %/g-C_3N_4 and of the same sample recovered after 6 h of irradiation under the conditions reported above are shown in Figure S5, indicating a very good stability of the composite after the photocatalytic test. To get a further insight into the microscopic mechanism underlying the HER behavior, we performed transient absorption spectroscopy (TAS) measurements on the composites.

Differential transmission (DT/T) measurements were performed on thin films of different $PhBz_2GeBr_{4/g\text{-}C_3N4}$ composites on quartz substrate, which we exploit to probe carrier dynamics. Note that, oftentimes, the technique is referred to as TAS since DT/T and absorption are linked and can be converted into each other. DT/T allows us to detect the changes in optical absorption that are induced by ultrafast laser pulses, in our case constituted by 100 fs-long pulses that are 320 nm in

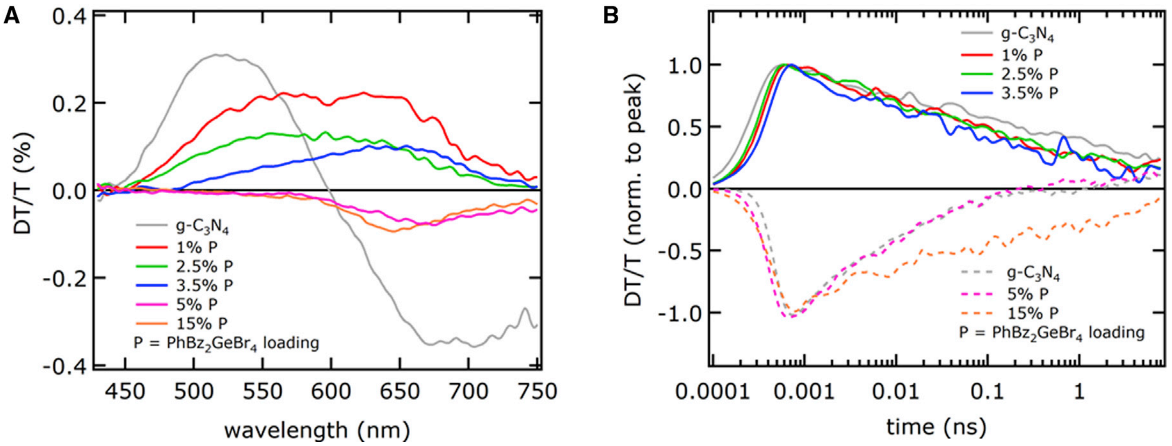

Figure 5. Transient absorption spectroscopy study of $PhBz_2GeBr_4$/g-C_3N_4 composites

Results of differential transmission on thin films of g-C_3N_4 with different $PhBz_2GeBr_4$ loadings, with excitation wavelength 320 nm.

(A) Differential transmission spectra integrated over time.

(B) Time decays of differential transmission signals integrated over wavelength, normalized for clear comparison, with bleaching features (positive) represented as solid lines, and negative bands (photoinduced absorption) depicted as dashed lines.

wavelength. DT/T is then determined with a pump-and-probe technique where fs supercontinuum white pulses are delayed with respect to pump pulses with an adjustable delay line (see the experimental section in the supplemental information). From the results collected in Figure 5, two broad DT/T features are visible in g-C_3N_4 sample (gray line): a positive bleaching band around 500 nm, resulting from filling of the excited state, and a negative band, which peaked around 650 nm, that can be associated with photoinduced absorption from the excited state. When adding $PhBz_2GeBr_4$ from 1 to 3.5 wt %, the 500 nm-bleaching band is gradually replaced by a lower-energy one centered around 650 nm. In contrast, for % wt >5, a photoinduced band, similar to pure g-C_3N_4, is partially recovered.

The simultaneous fading of photoinduced signal/high-energy bleaching and the appearance of low-energy bleaching in DT/T spectra may be interpreted as evidence for defect filling at the g-C_3N_4/perovskite interface, possibly due to charge transfer. Both PL band structure and bleaching in DT/T can be therefore correlated with partial charge transfer to g-C_3N_4, which happens to be more efficient for intermediate compositions (1%–3.5% loading), leading to better photocatalytic performance. At variance with this, samples with above 5 wt % have PL and DT/T spectra more similar to g-C_3N_4, an indication that most photoexcited electrons are kept in g-C_3N_4 states, leading to either radiative recombination or to photoinduced absorption. Such a trend with respect to perovskite loading is compatible with what has been already observed in a Bi-based perovskite composite for photocatalysis.[34] The trend of both a DT/T bleaching feature and hydrogen production rate can be linked with defect filling at the g-C_3N_4 /perovskite interfaces but with a disclaimer: the defect states involved in absorption process, affecting DT/T dynamics, are substantially not taking part in PL emission, which results instead from g-C_3N_4 states. In fact, the results of time-resolved PL measurements show that the PL lifetime of g-C_3N_4/perovskite compounds is not different from that of g-C_3N_4 and is not varying with perovskite loading, despite pure perovskite showing a much shorter lifetime than g-C_3N_4 compounds (see Figure 3D).

Computational modeling

The origin of the reported results was further investigated from an atomistic perspective with the aim of understanding (1) the outstanding water stability of

CellPress
OPEN ACCESS

Table 1. Calculated values of E_f(bulk) and $E_f(V_A)$ (cf. main text for definitions) for the considered A_2GeX_4 perovskites

Perovskite	E_f(bulk)	$E_f(V_A)$	Water stable?
PEA_2GeBr_4	−4.15	3.98	no
$BrPEA_2GeBr_4$	−4.12	4.18	no
$(BPEA)_2GeI_4$	−4.65	4.92	yes
$(BPEA)_2GeBr_4$	−4.80	5.26	yes

the Ge-based perovskites synthetized in this work, and (2) the efficient photocatalytic production of H_2 when the perovskites are used in a composite with g-C_3N_4 (see Note S1). In addition, computational modeling was extended to also include the iodide-analogous phase, namely $PhBz_2GeI_4$, to determine its possible application in photocatalysis. As a matter of fact, the presence of iodide can red shift the band gap and provide a more effective charge transfer in the heterostructure.[20,24,26] However, iodide-based materials are known to be less stable than the bromide counterparts.

To fulfill the first goal, we calculated the bulk formation energies E_f(bulk) of the water-stable Ge-based perovskites, and we compared them with those of previously synthetized materials, bearing different A-site spacer cations, which were found to dissolve in aqueous environment.[24] Since no experimental crystallographic structure is currently available for $PhBz_2GeI_4$ and $PhBz_2GeBr_4$, we consider the perovskites with BPEA, which differs from PhBz only for an extra CH_2 between the aromatic ring and the ammonium moiety.[25] $BPEA_2GeI_4$ and $BPEA_2GeBr_4$ were modeled starting from the analogous $BPEA_2PbI_4$ as described in the supplemental information. We consider, for our comparison, PEA_2GeI_4 and $BrPEA_2GeBr_4$, which have been synthetized and characterized in Chiara et al.[24] The details of the structural models are reported in the supplemental information.

From the following reaction

$$2AX + GeX_2 \rightarrow A_2GeX_4, \quad \text{(Equation 1)}$$

where A = PEA, BrPEA, and BPEA and X = Br and I, we define E_f(bulk) as follows:

$$E_f(\text{bulk}) = E(A_2GeX_4) - E(AX) - E(GeX_2). \quad \text{(Equation 2)}$$

In Equation 2, $E(A_2GeX_4)$, $E(AX)$, and $E(GeX_2)$ are the total energies of A_2GeX_4, AX, and GeX_2, which are calculated from their respective atomistic models (cf. Note S1). Results collected in Table 1 clearly indicate that perovskites bearing BPEA as spacer A-site cation are substantially more stable than the others, with formation energies being up to 0.7 eV lower. Such a larger stability may be ascribed to the enhanced vdW interactions available within the A-cation layers separating the inorganic frameworks when using the larger BPEA molecule. In order to further verify this consideration, we calculate for each studied material the formation energy of a neutral A vacancy, V_A, which is defined as

$$E_f(V_A) = E(V_A) - E(A_2GeX_4) - E(A), \quad \text{(Equation 3)}$$

where $E(V_A)$ is the total energy of the perovskite model with a missing A molecule and $E(A)$ the total energy of an isolated A molecule. From Table 1, we evince that the energy associated with the removal of an A cation from the bulk perovskite is remarkably higher (up to 1 eV) for BPEA when compared with the other systems, thus clearly indicating that intra-layer vdW interactions sizably stabilize BPEA perovskites. Furthermore, the higher solvation Gibbs free energies calculated for BPEA and PhBz (cf. Table S4) indicate a reduced tendency of these cations to dissolve in water when

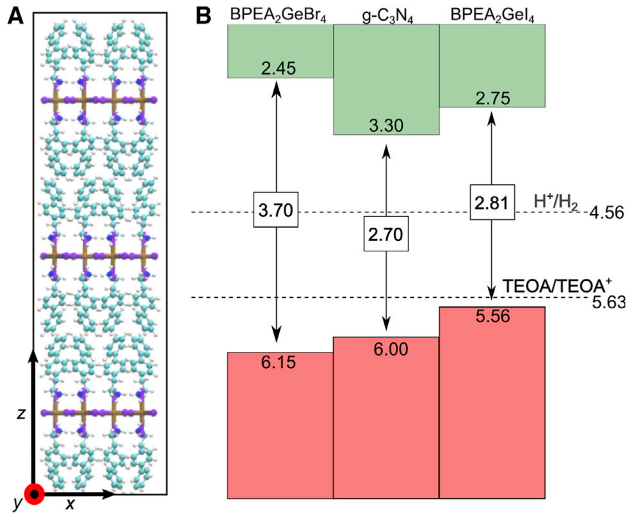

Figure 6. Computational modeling of surface termination and band alignment for $BPEA_2GeI_4$ and $BPEA_2GeBr_4$

(A) Stick-and-ball representation of the atomistic model for the (BPEA)I-terminated (100) slab of $BPEA_2GeI_4$ perovskite. Ge atoms are depicted in ochre, I in violet, C in cyan, N in blue, and H in white.

(B) Valence band (VB) and conduction band (CB) edges of g-C_3N_4, $BPEA_2GeI_4$, and $BPEA_2GeBr_4$ referred to the vacuum level. The energy level of standard hydrogen electrode calculated in Romani et al.[9] and the TEOA/TEOA redox level are reported as dashed lines.

compared with the cations employed in previous work.[24] We note that such a result is in line with a previous observation of the increased water stability observed when replacing methylammonium with dimethylammonium in tin perovskites.[31,37]

To study the beneficial effect on the photocatalytic H_2 production of the $(BPEA)_2GeX_4$ perovskites in a composite with g-C_3N_4, we investigated their band alignment. To this end, we constructed atomistic models of the surface of these materials. When considering the (100) surface of $(BPEA)_2GeX_4$, we find that the most stable termination is the stoichiometric (BPEA)X-terminated one, with the BPEA cations pointing their hydrophobic biphenyl moieties toward the surface (cf. Figure 6A for the (BPEA)I terminated surface of $(BPEA)_2GeI_4$), while the ammonium moieties interact with the subsurface inorganic chain. This termination features a surface energy as small as 0.005 eV/Å^2 (cf. supplemental information for details of the calculations).

Then, we employed advanced electronic-structure calculations to evaluate the band gap of the materials and align the band edges with respect to the vacuum level (cf. Note S1 and Tables S2 and S3). The band alignment for $(BPEA)_2GeI_4$ and $(BPEA)_2GeBr_4$ is reported in Figure 6B along with that previously calculated in Romani et al.[9] for g-C_3N_4. The band edges of $(BPEA)_2GeI_4$ are found be favorably aligned with respect to those of g-C_3N_4, thus promoting the transfer of photogenerated charges in a type 2 heterojunction, which could also improve the carrier lifetime. At variance with this, $(BPEA)_2GeBr_4$, which features a larger band gap (3.7 versus 2.81 eV), has a valence band edge at an energy 0.15 eV lower than g-C_3N_4, providing a type I heterojunction. This implies that hole transfer from g-C_3N_4 to the perovskites might be subject to a small energy barrier, which may reduce the efficiency of the composite.

The results of the computational modeling have put in prominence two main results: (1) confirmation of the water stability for $BPEA_2GeBr_4$ together with a beneficial band

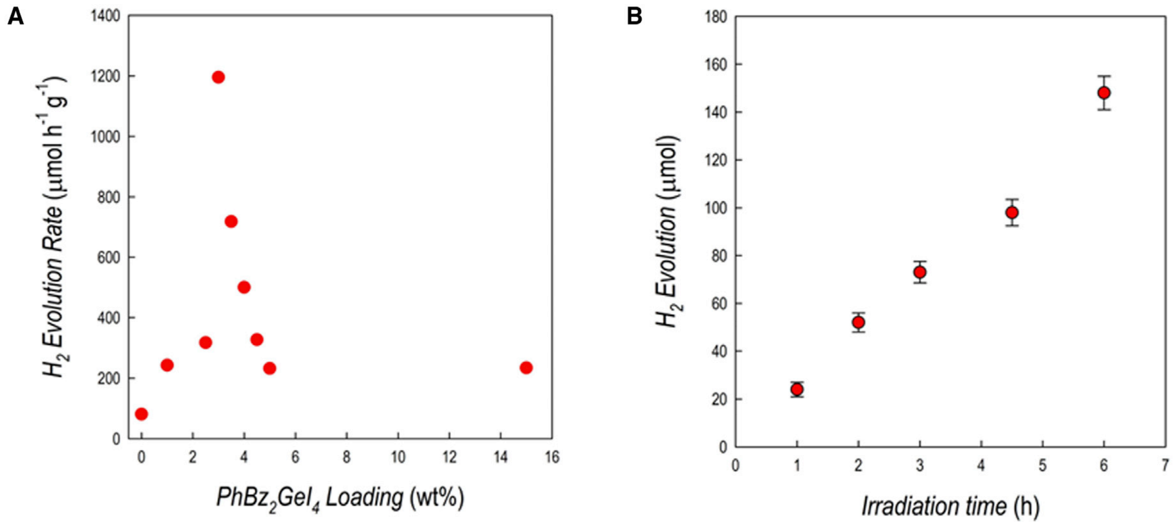

Figure 7. Hydrogen photogeneration performance of $PhBz_2Ge_2I_4$/g-C_3N_4 composites

(A) Hydrogen evolution rates for $PhBz_2Ge_2I_4$/g-C_3N_4 composites, 6 h irradiation, RSD <15% (n = 3).

(B) Hydrogen evolution profile over irradiation time for the 3 wt % $PhBz_2Ge_2I_4$/g-C_3N_4 composite, RSD <10% (n = 3). Conditions: 1 g L^{-1} catalyst, 10% v/v TEOA, 3 wt % Pt, simulated solar light (Xenon lamp, 500 W m^{-2}, 300–800 nm, IR-treated soda-lime glass UV outdoor filter).

alignment with g-C_3N_4, as demonstrated by the above reported hydrogen photogeneration experiments, and (2) prediction of good water stability by $BPEA_2GeI_4$ (even though it has lower E_f(bulk) and $E_f(V_A)$ with respect to $BPEA_2GeBr_4$) and possible superior photocatalytic performance due to a better band alignment with respect to the bromide-containing counterpart.

$PhBz_2GeI_4$ hydrogen generation experiments

To test this last computational evidence, we synthesized the $PhBz_2GeI_4$ perovskite and a series of composites with g-C_3N_4 (1%, 2.5%, 3%, 3.5%, 4%, 4.5%, 5%, and 15% of perovskite loading). Figures S6 and S7 report the XRD and absorbance data on the composite. We used the same approach we employed for $PhBz_2GeBr_4$ in testing the air and water stability of $PhBz_2GeI_4$. Figure S8 also shows good air and water stabilities for the iodide-containing perovskites according to the computational prediction. However, the leaching test revealed an amount of Ge in the solution of about 40% already after 4 h of stirring. This value remains constant after 8 and 16 h of stirring (42% and 43%), suggesting the possible occurrence of a solubility equilibrium that then keeps the perovskite unchanged in the solution according to the XRD diffraction after recovering the powder (Figure S8). Substantially the same leaching (44%) was noticed also under photocatalytic conditions, turning in line with the overall good photocatalytic performance of this composite, described hereafter. Even though the $PhBz_2GeI_4$ is less stable in water, which also agrees with the calculated values of E_f(bulk), $E_f(V_A)$, we performed a thorough investigation of the hydrogen photogeneration efficiency. Figure 7A reports the HER as a function of perovskite loading for the $PhBz_2GeI_4$/g-C_3N_4 composites, while Figure 7B shows the kinetics of the hydrogen photogeneration for the best-performing composite from Figure 7A, namely 3 wt % $PhBz_2GeI_4$/g-C_3N_4. For this system, the AQY% is 2.8%, which is lower with respect to the best-performing $PhBz_2GeBr_4$/g-C_3N_4 composite. The higher HER with a lower AQY (calculated as the percent ratio H_2 moles/incident photons moles) is in line with the presence of iodide extending the absorption in the visible part of the spectrum, allowing it to harness more photons. The overall result for the HER of $PhBz_2$

$GeBr_4/g\text{-}C_3N_4$ composites is observed to be higher, but the AQY is not, for a diverse absorptivity of the specimens.

The HER for the $PhBz_2GeI_4/g\text{-}C_3N_4$ composites is higher with respect to the $PhBz_2GeBr_4/g\text{-}C_3N_4$ composites, reaching a value of about 1,200 μmol g^{-1} h^{-1}, again with a synergic effect with respect to pure carbon nitride (81 μmol g^{-1} h^{-1}), and of about 600 times with respect to pure $PhBz_2GeI_4$ (2 μmol g^{-1} h^{-1}). The maximum HER is found at 3 wt %, very close to the value of 2.5 wt % found for the $PhBz_2GeBr_4/g\text{-}C_3N_4$ series. Again, according to the computational modeling, we could confirm the better band alignment of this perovskite with $g\text{-}C_3N_4$, leading to a better performing heterostructure. While this composite proved to be less stable than the bromide counterpart, these results pave the way to further explore this system by improving its stability in water through encapsulations strategies. It is also interesting to confirm the trend as a function of loading, which peaks around 3%, in agreement with the TAS results reported above and with the previous results we provided on the $Cs_3Bi_2Br_9/g\text{-}C_3N_4$ composites.[34]

Finally, to evaluate the potential of the two best-performing composites for practical photocatalytic applications, 2.5 wt % $PhBz_2GeBr_4/g\text{-}C_3N_4$ and 3 wt % $PhBz_2GeI_4/g\text{-}C_3N_4$ were tested for H_2 evolution from aqueous solutions of glucose and starch, chosen as representative biomass derivatives. Under optimized conditions, H_2 formation was appreciable for both catalysts (Table S1) and was still higher using the iodine-based composite and with a greater H_2 yield in the presence of the monosaccharide, which exhibits faster mass transfer kinetics compared with the branched-skeleton polysaccharide.[36] The observed HERs are clearly higher than those of the control samples (no catalysts, <0.008 μmol h^{-1}), demonstrating the effectiveness of such new composites for H_2 photogeneration also from biomasses in solution.

In conclusion, we report on the realization of an intrinsically water stable 2D Ge-based halide perovskite, a material that can sustain suspension in water for several hours. The strategy employed for the realization of such innovative system foresees the use of an extended π-conjugated organic cation (phenylbenzylammonium), which, through intra-layer vdW interactions, sizably stabilizes the resulting bulk perovskite. The material has been tested therefore for simulated solar-light-induced hydrogen evolution from water and aqueous solutions of glucose and starch in combination with a partnering material, which induces the formation of an active heterojunction, leading to very promising HERs up to 1,200 μmol g^{-1} h^{-1}. This is an outstanding result considering the seminal exploitation of a Ge perovskite for such a purpose and provide a proof of concept for the use of 2D Ge-based MHPs. Most importantly, these findings contribute to expand the rationale behind the intelligent design of intrinsically water-stable MHP phases. The achievement of such an understating would massively impact MHP-based photocatalytic applications but also alternative optoelectronic innovations based on classes of materials such as LED, photovoltaics (PV), and detectors, whose technological declination has been delayed, among other factors, by moisture-exposure weakness of the active material.

EXPERIMENTAL PROCEDURES

Resource availability

Lead contact

Further information and requests for resources and reagents should be directed to and will be fulfilled by the lead contact, Lorenzo Malavasi (lorenzo.malavasi@unipv.it), and the other corresponding authors (andrea.listorti@uniba.it and francesco.ambrosio@unibas.it).

Materials availability

All unique or stable reagents generated in this study are available from the lead contact with a completed materials transfer agreement.

Data and code availability

The authors declare that the data supporting the findings of this study are available within the article and the supplemental information.

Sample preparation

Bulk g-C_3N_4 has been synthesized from the polymerization of DCD (NH_2C(=NH) NHCN, Aldrich, 99%) by the following thermal treatment (under N_2 flux): heating (1 °C/min) to 550°C, isothermal step for 4 h followed by cooling to room temperature (10 °C/min). Synthesis has been carried out in a partially closed alumina crucible. $PhBz_2GeX_4$ (X = I and Br) perovskites have been prepared by dissolving GeO_2 in HX under stirring and under nitrogen flux. The $PhBz_2GeX_4$/g-C_3N_4 composites have been prepared by adding to the DMF solution containing the perovskites the proper amount of g-C_3N_4 prepared as described above.

Sample characterization

The crystal structure of the samples has been characterized by room-temperature Cu-radiation XRD acquired with a Bruker D8 diffractometer. Diffuse reflectance spectroscopy (DRS) spectra were acquired in the wavelength range 300–800 nm directly on the powders by using a Jasco V-750 spectrophotometer, equipped with an integrating sphere (Jasco ISV-922). Microstructural characterization of the samples was made using a high-resolution SEM (TESCAN Mira 3) operated at 25 kV. Elemental mapping has been performed on the best-performing and stable composite (cf. section "$PhBz_2GeBr_4$ hydrogen generation experiments"), namely 2.5 wt % $PhBz_2GeBr_4$/g-C_3N_4, and the data are reported in Figure S9. Due to the very low amount of metal present in the sample, clear distribution has been obtained only for the Br, C, and N elements, indicating a good distribution of the perovskite in the carbon nitride matrix.

The PL measurements were recorded by means of a Fluorolog-3 spectrofluorometer (HORIBA Jobin-Yvon) equipped with a 450 W xenon lamp as the exciting source and double grating excitation and emission monochromators. All optical measurements were performed at room temperature on powder dispersed samples as obtained from the synthesis without any size sorting treatment. The PL emission spectra were recorded by using an excitation wavelength of 375 nm.

The DT/T was measured on the composites dispersed in Nafion matrix by exciting them with a pulsed laser source (100 fs pulses at 320 nm wavelength) obtained from a kHz regenerative amplifier and an optical parametric oscillator (Coherent Libra and Light Conversion Topas 800). DT/T is then determined with a pump-and-probe technique, where the sample is excited also with fs supercontinuum white pulses, obtained by focusing the fundamental output regenerative amplifier (800 nm) onto a sapphire plate, whose delay with respect to pump pulses is controlled with an adjustable mechanical delay line. DT/T spectra are finally obtained from signal and reference spectra acquired with a couple of CMOS grating spectrometers (Ultrafast Systems Helios).

Hydrogen evolution experiments

H_2 evolution experiments were conducted in distilled water containing 10% (v/v) TEAO (Aldrich, ≥99%), irradiated in Pyrex glass containers (28 mL capacity, 21 mL

sample). After the addition of the catalyst (1 g L^{-1}), the sample was deoxygenated by Ar bubbling (20 min) to obtain anoxic conditions and irradiated under magnetic stirring for 6 h.

Chloroplatinic acid (H_2PtCl_6, 38% Pt basis), used as precursor for metallic Pt, was from Sigma-Aldrich. Since Pt is *in situ* photodeposited on the catalyst surface, after Ar bubbling, a small volume from a 15 g L^{-1} H_2PtCl_6 aqueous solution was added, using a 10–100 μL micropipette, to the catalyst suspension (1 g L^{-1}) directly in the photoreactor. The latter was closed with sleeve stopper septa and was irradiated, as described in the following, achieving simultaneous Pt deposition and H_2 production. Irradiation was performed under simulated solar light (1,500 W Xenon lamp, 300–800 nm) using a Solar Box 1500e (CO.FO.ME.GRA S.r.l., Milan, Italy) set at a power factor 500 W m^{-2} and equipped with UV outdoor filter made of infrared (IR)-treated soda-lime glass. Triplicate photoproduction experiments were performed on all samples. The headspace-evolved gas was quantified by gas chromatography coupled with thermal conductivity detection (GC-TCD). The results obtained in terms of H_2 evolution rate are expressed in the paper as μmoles of gas per gram of catalyst per hour (μmoles g^{-1} h^{-1}). XRD measurements on spent catalysts have been done by filtering the suspensions and recovering the powder, which underwent diffraction measurements.

Metal leaching tests

The leaching tests were performed by dispersion of the powders in distilled water under magnetic stirring for 4, 8, 16, and 24 h. Then, the suspension was filtered on 0.2 μm nylon membrane, and the amount of tin in solution was determined by ICP-OES analysis after acidification (1% v/v ultrapure nitric acid).

SUPPLEMENTAL INFORMATION

Supplemental information can be found online at https://doi.org/10.1016/j.xcrp.2022.101214.

ACKNOWLEDGMENTS

F.A., E.M., and F.D.A. acknowledge support from the Ministero Istruzione dell'Università e della Ricerca (MIUR) and the University of Perugia through the program "Dipartimenti di Eccellenza 2018–2022" (grant AMIS) and from the European Union's Horizon 2020 research and innovation program under grant agreement no. 764047 of the Espresso project. This work has been financed by the Research Fund for the Italian Electrical Systemunder the contract agreement between RSE S.p.A. and the Ministry of Economic Development - General Directorate for the Electricity Market, Renewable Energy and Energy Efficiency, Nuclear Energy, in compliance with the Decree of April 16th, 2018. A.S. was supported by PON "Ricerca e Innovazione" 2014–2020 FSE, project AIM1809115-2. A.L. acknowledges support from Puglia regional council (grant name: Perseo, CUP: H95F20000890003).

AUTHOR CONTRIBUTIONS

L.R., M.M.-L., C.C., and C.T. performed photocatalysis experiments; A.S. and A.P. supervised photocatalysis experiments and analyzed the data; M.M. carried out structural characterization; V.A., S.C., A.M., and A.L. performed part of the optical characterization and XPS experiments; F.A., E.M., D.R., and F.D.A. performed computational modeling work and analyzed the data, R.P., F.P., A.S., and M.S. carried out TAS measurements and related data analysis; and L.M. devised the work.

CellPress
OPEN ACCESS

The manuscript was written by L.M., A.L., and F.A. All authors have approved the final version of the manuscript.

DECLARATION OF INTERESTS

The authors declare no competing interests.

Received: April 29, 2022
Revised: November 22, 2022
Accepted: December 8, 2022
Published: January 5, 2023

REFERENCES

1. Romani, L., and Malavasi, L. (2020). Solar-driven hydrogen generation by metal halide perovskites: materials, approaches, and mechanistic view. ACS Omega *5*, 25511–25519. https://doi.org/10.1021/acsomega.0c03829.

2. Huang, H., Pradhan, B., Hofkens, J., Roeffaers, M.B.J., and Steele, J.A. (2020). Solar-driven metal halide perovskite photocatalysis: design, stability, and performance. ACS Energy Lett. *5*, 1107–1123. https://doi.org/10.1021/acsenergylett.0c00058.

3. Bresolin, B.-M., Park, Y., and Bahnemann, D. (2020). Recent progresses on metal halide perovskite-based material as potential photocatalyst. Catalysts *10*, 709. https://doi.org/10.3390/catal10060709.

4. Huynh, K.A., Nguyen, D.L.T., Nguyen, V., Vo, D.N., Trinh, Q.T., Nguyen, T.P., Kim, S.Y., and Le, Q.V. (2020). Halide perovskite photocatalysis: progress and perspectives. J. Chem. Technol. Biotechnol. *95*, 2579–2596. https://doi.org/10.1002/jctb.6342.

5. Armenise, V., Colella, S., Fracassi, F., and Listorti, A. (2021). Lead-free metal halide perovskites for hydrogen evolution from aqueous solutions. Nanomaterials *11*, 433. https://doi.org/10.3390/nano11020433.

6. Bhattacharjee, S., Chaudhary, S.P., and Bhattacharyya, S. (2019). Lead-free metal halide perovskite nanocrystals for photocatalysis in water. Preprint at ChemRxiv. https://doi.org/10.26434/chemrxiv.9794270.v1.

7. Bresolin, B.-M., Sgarbossa, P., Bahnemann, D.W., and Sillanpää, M. (2020). Cs3Bi2I9/g-C3N4 as a new binary photocatalyst for efficient visible-light photocatalytic processes. Separ. Purif. Technol. *251*, 117320. https://doi.org/10.1016/j.seppur.2020.117320.

8. Romani, L., Bala, A., Kumar, V., Speltini, A., Milella, A., Fracassi, F., Listorti, A., Profumo, A., and Malavasi, L. (2020). PEA2SnBr4: a water-stable lead-free two-dimensional perovskite and demonstration of its use as a co-catalyst in hydrogen photogeneration and organic-dye degradation. J. Mater. Chem. C *8*, 9189–9194. https://doi.org/10.1039/D0TC02525A.

9. Romani, L., Speltini, A., Ambrosio, F., Mosconi, E., Profumo, A., Marelli, M., Margadonna, S., Milella, A., Fracassi, F., Listorti, A., et al. (2021). Water-stable DMASnBr3 lead-free perovskite for effective solar-driven photocatalysis. Angew. Chem. Int. Ed. Engl. *60*, 3611–3618. https://doi.org/10.1002/anie.202007584.

10. Dai, Y., and Tüysüz, H. (2019). Lead-free Cs3Bi2Br9 perovskite as photocatalyst for ring-opening reactions of epoxides. ChemSusChem *12*, 2587–2592. https://doi.org/10.1002/cssc.201900716.

11. Bresolin, B.-M., Balayeva, N.O., Granone, L.I., Dillert, R., Bahnemann, D.W., and Sillanpää, M. (2020). Anchoring lead-free halide Cs3Bi2I9 perovskite on UV100–TiO2 for enhanced photocatalytic performance. Sol. Energy Mater. Sol. Cell. *204*, 110214. https://doi.org/10.1016/j.solmat.2019.110214.

12. Luo, J., Zhang, W., Yang, H., Fan, Q., Xiong, F., Liu, S., Li, D., and Liu, B. (2021). Halide perovskite composites for photocatalysis: a mini review. EcoMat *3*. https://doi.org/10.1002/eom2.12079.

13. Pan, S., Li, J., Wen, Z., Lu, R., Zhang, Q., Jin, H., Zhang, L., Chen, Y., and Wang, S. (2022). Halide perovskite materials for photo(Electro) Chemical applications: dimensionality, heterojunction, and performance. Adv. Energy Mater. *12*. 2004002. https://doi.org/10.1002/aenm.202004002.

14. Yuan, J., Liu, H., Wang, S., and Li, X. (2021). How to apply metal halide perovskites to photocatalysis: challenges and development. Nanoscale *13*, 10281–10304. https://doi.org/10.1039/D0NR07716J.

15. Han, X., Liu, P., Ran, R., Wang, W., Zhou, W., and Shao, Z. (2022). Non-metal fluorine doping in Ruddlesden–Popper perovskite oxide enables high-efficiency photocatalytic water splitting for hydrogen production. Mater. Today Energy *23*, 100896. https://doi.org/10.1016/j.mtener.2021.100896.

16. He, J., Liu, P., Ran, R., Wang, W., Zhou, W., and Shao, Z. (2022). Single-atom catalysts for high-efficiency photocatalytic and photoelectrochemical water splitting: distinctive roles, unique fabrication methods and specific design strategies. J. Mater. Chem. *10*, 6835–6871. https://doi.org/10.1039/D2TA00835A.

17. Park, S., Chang, W.J., Lee, C.W., Park, S., Ahn, H.-Y., and Nam, K.T. (2017). Photocatalytic hydrogen generation from hydriodic acid using methylammonium lead iodide in dynamic equilibrium with aqueous solution. Nat. Energy *2*, 16185. https://doi.org/10.1038/nenergy.2016.185.

18. Zheng, C., and Rubel, O. (2019). Unraveling the water degradation mechanism of CH3NH3PbI3. J. Phys. Chem. C *123*, 19385–19394. https://doi.org/10.1021/acs.jpcc.9b05516.

19. Christians, J.A., Miranda Herrera, P.A., and Kamat, P.V. (2015). Transformation of the excited state and photovoltaic efficiency of CH3NH3PbI3 perovskite upon controlled exposure to humidified air. J. Am. Chem. Soc. *137*, 1530–1538. https://doi.org/10.1021/ja511132a.

20. Li, X., Hoffman, J.M., and Kanatzidis, M.G. (2021). The 2D halide perovskite rulebook: how the spacer influences everything from the structure to optoelectronic device efficiency. Chem. Rev. *121*, 2230–2291. https://doi.org/10.1021/acs.chemrev.0c01006.

21. Pisanu, A., Speltini, A., Quadrelli, P., Drera, G., Sangaletti, L., and Malavasi, L. (2019). Enhanced air-stability of Sn-based hybrid perovskites induced by dimethylammonium (DMA): synthesis, characterization, aging and hydrogen photogeneration of the MA1−xDMAxSnBr3 system. J. Mater. Chem. C *7*, 7020–7026. https://doi.org/10.1039/C9TC01743G.

22. Ju, D., Zheng, X., Liu, J., Chen, Y., Zhang, J., Cao, B., Xiao, H., Mohammed, O.F., Bakr, O.M., and Tao, X. (2018). Reversible band gap narrowing of Sn-based hybrid perovskite single crystal with excellent phase stability. Angew. Chem. Int. Ed. Engl. *57*, 14868–14872. https://doi.org/10.1002/anie.201810481.

23. Hong, Z., Chong, W.K., Ng, A.Y.R., Li, M., Ganguly, R., Sum, T.C., and Soo, H.S. (2019). Hydrophobic metal halide perovskites for visible-light photoredox C−C bond cleavage and dehydrogenation catalysis. Angew. Chem. Int. Ed. Engl. *58*, 3456–3460. https://doi.org/10.1002/anie.201812225.

24. Chiara, R., Morana, M., Boiocchi, M., Coduri, M., Striccoli, M., Fracassi, F., Listorti, A., Mahata, A., Quadrelli, P., Gaboardi, M., et al. (2021). Role of spacer cations and structural distortion in two-dimensional germanium halide perovskites. J. Mater. Chem. C *9*, 9899–9906. https://doi.org/10.1039/D1TC02394B.

25. Venkatesan, N.R., Mahdi, A., Barraza, B., Wu, G., Chabinyc, M.L., and Seshadri, R. (2019). Enhanced yield-mobility products in hybrid halide Ruddlesden–Popper compounds with aromatic ammonium spacers. Dalton Trans. *48*,

14019–14026. https://doi.org/10.1039/C9DT03074C.

26. Malavasi, L., Morana, M., and Chiara, R. (2021). Germanium-based halide perovskites: materials, properties, and applications. ChemPlusChem *86*, 879–888. https://doi.org/10.1002/cplu.202100191.

27. Chen, M., Ju, M.-G., Garces, H.F., Carl, A.D., Ono, L.K., Hawash, Z., Zhang, Y., Shen, T., Qi, Y., Grimm, R.L., et al. (2019). Highly stable and efficient all-inorganic lead-free perovskite solar cells with native-oxide passivation. Nat. Commun. *10*, 16. https://doi.org/10.1038/s41467-018-07951-y.

28. Prabhakaran, K., and Ogino, T. (1995). Oxidation of Ge(100) and Ge(111) surfaces: an UPS and XPS study. Surf. Sci. *325*, 263–271. https://doi.org/10.1016/0039-6028(94)00746-2.

29. Wagner, C.D., Zatko, D.A., and Raymond, R.H. (1980). Use of the oxygen KLL Auger lines in identification of surface chemical states by electron spectroscopy for chemical analysis. Anal. Chem. *52*, 1445–1451. https://doi.org/10.1021/ac50059a017.

30. Meggiolaro, D., Ricciarelli, D., Alasmari, A.A., Alasmary, F.A.S., and De Angelis, F. (2020). Tin versus lead redox chemistry modulates charge trapping and self-doping in tin/lead iodide perovskites. J. Phys. Chem. Lett. *11*, 3546–3556. https://doi.org/10.1021/acs.jpclett.0c00725.

31. Ricciarelli, D., Kaiser, W., Mosconi, E., Wiktor, J., Ashraf, M.W., Malavasi, L., Ambrosio, F., and De Angelis, F. (2022). Reaction mechanism of photocatalytic hydrogen production at water/tin halide perovskite interfaces. ACS Energy Lett. *7*, 1308–1315. https://doi.org/10.1021/acsenergylett.2c00122.

32. Sharma, A., Varshney, M., Chae, K.H., and Won, S.O. (2018). Mechanistic investigations on emission characteristics from g-C3N4, g-C3N4@Pt and g-C3N4@Ag nanostructures using X-ray absorption spectroscopy. Curr. Appl. Phys. *18*, 1458–1464. https://doi.org/10.1016/j.cap.2018.08.019.

33. Yuan, Y., Zhang, L., Xing, J., Utama, M.I.B., Lu, X., Du, K., Li, Y., Hu, X., Wang, S., Genç, A., et al. (2015). High-yield synthesis and optical properties of g-C3N4. Nanoscale *7*, 12343–12350. https://doi.org/10.1039/C5NR02905H.

34. Romani, L., Speltini, A., Dibenedetto, C.N., Listorti, A., Ambrosio, F., Mosconi, E., Simbula, A., Saba, M., Profumo, A., Quadrelli, P., et al. (2021). Experimental strategy and mechanistic view to boost the photocatalytic activity of $Cs_3Bi_2Br_9$ lead-free perovskite derivative by $g\text{-}C_3N_4$ composite engineering. Adv. Funct. Mater. *31*, 2104428. https://doi.org/10.1002/adfm.202104428.

35. Cao, S., Low, J., Yu, J., and Jaroniec, M. (2015). Polymeric photocatalysts based on graphitic carbon nitride. Adv. Mater. *27*, 2150–2176. https://doi.org/10.1002/adma.201500033.

36. Speltini, A., Romani, L., Dondi, D., Malavasi, L., and Profumo, A. (2020). Carbon nitride-perovskite composites: evaluation and optimization of photocatalytic hydrogen evolution in Saccharides aqueous solution. Catalysts *10*, 1259. https://doi.org/10.3390/catal10111259.

37. Kaiser, W., Ricciarelli, D., Mosconi, E., Alothman, A.A., Ambrosio, F., and De Angelis, F. (2022). Stability of tin- versus lead-halide perovskites: Ab initio molecular dynamics simulations of perovskite/water interfaces. J. Phys. Chem. Lett. *13*, 2321–2329. https://doi.org/10.1021/acs.jpclett.2c00273.

Cell Reports Physical Science, Volume *4*

Supplemental information

Air- and water-stable and photocatalytically active germanium-based 2D perovskites by organic spacer engineering

Lidia Romani, Andrea Speltini, Rossella Chiara, Marta Morana, Clarissa Coccia, Costanza Tedesco, Vincenza Armenise, Silvia Colella, Antonella Milella, Andrea Listorti, Antonella Profumo, Francesco Ambrosio, Edoardo Mosconi, Riccardo Pau, Federico Pitzalis, Angelica Simbula, Damiano Ricciarelli, Michele Saba, Maria Medina-Llamas, Filippo De Angelis, and Lorenzo Malavasi

SUPPLEMENTAL INFORMATION

(a)

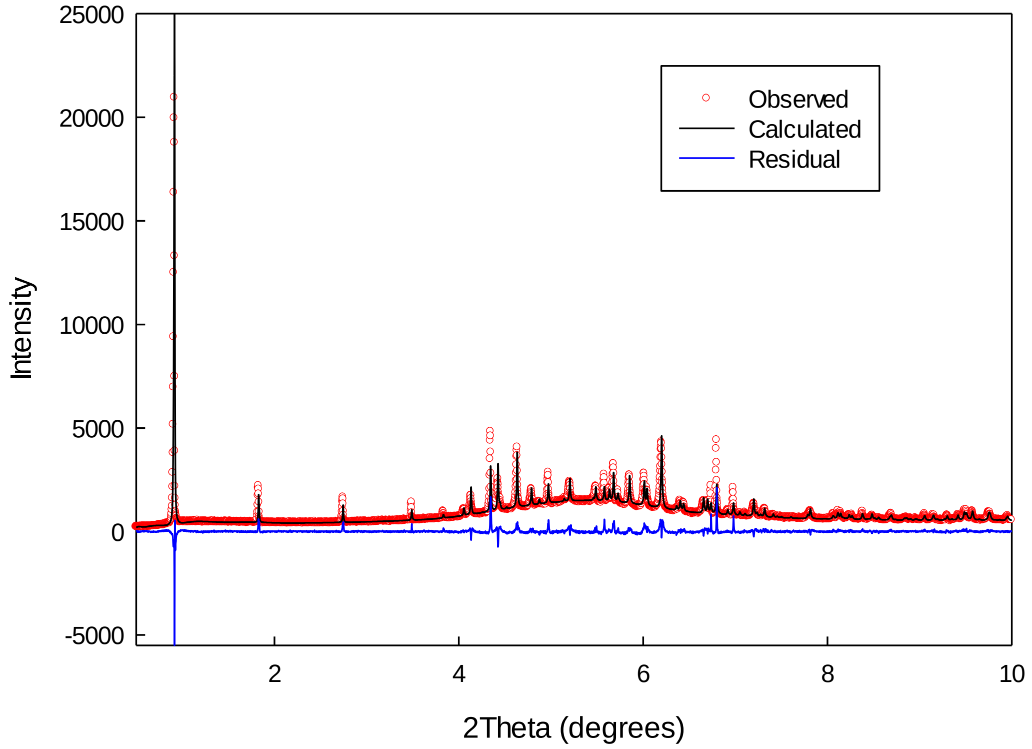

(b)

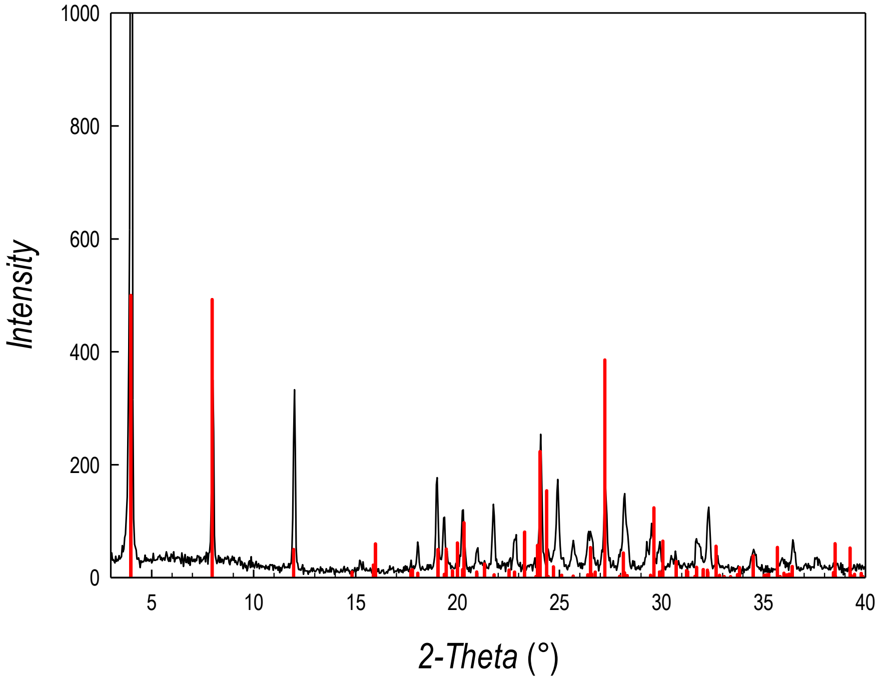

Figure S1. Structural properties of $PhBz_2GeBr_4$. Related to Figure 1. a) Indexed patter of $PhBz_2GeBr_4$ from synchrotron data (λ=0.3547); b) Diffraction pattern (Cu-alpha radiation) of $PhBz_2GeBr_4$ against the expected Bragg peaks of $BPEA_2PbI_4$ with actual lattice parameters obtained from the indexing procedure.

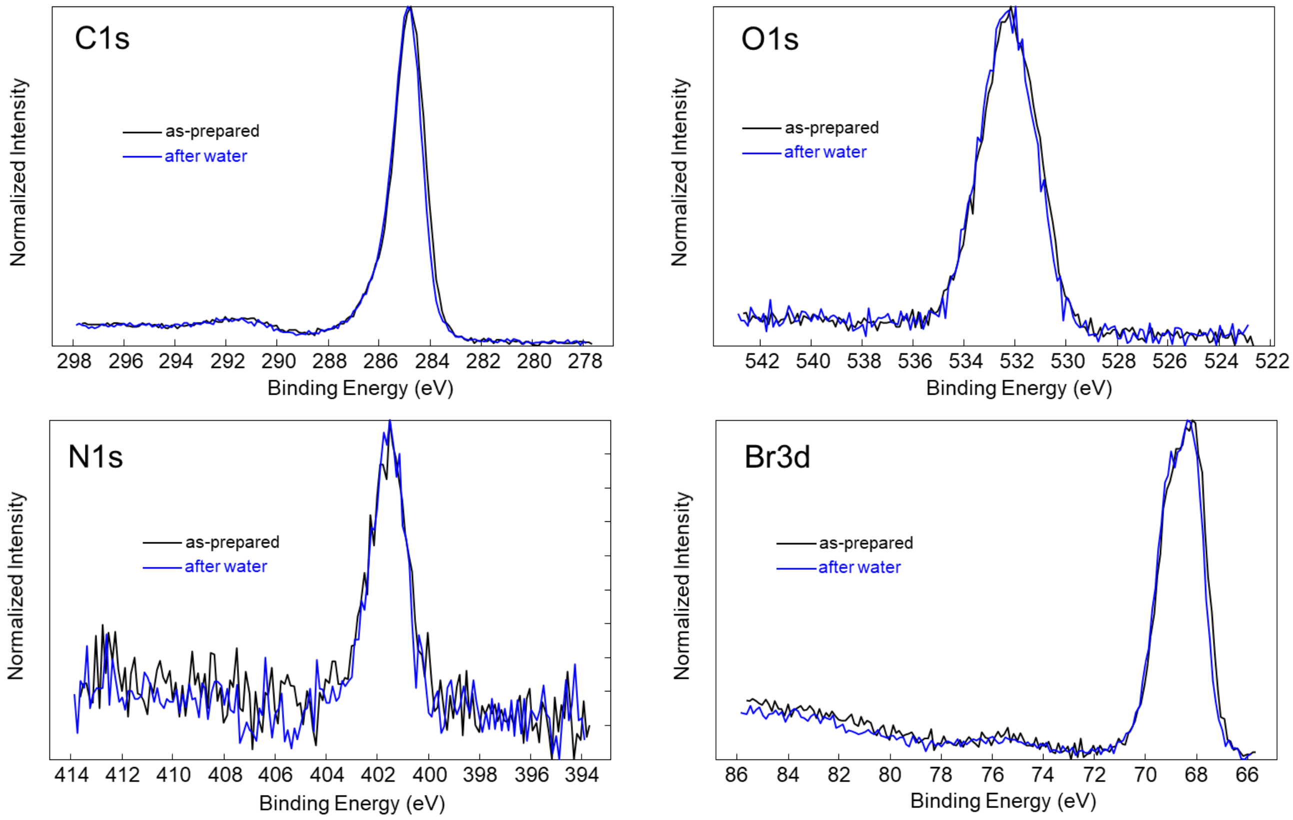

Figure S2. Photoelectron spectroscopy $PhBz_2GeBr_4$. Related to Figure 2. XPS spectra of as-prepared $PhBz_2GeBr_4$ (black), and after 24 h water treatment (blue).

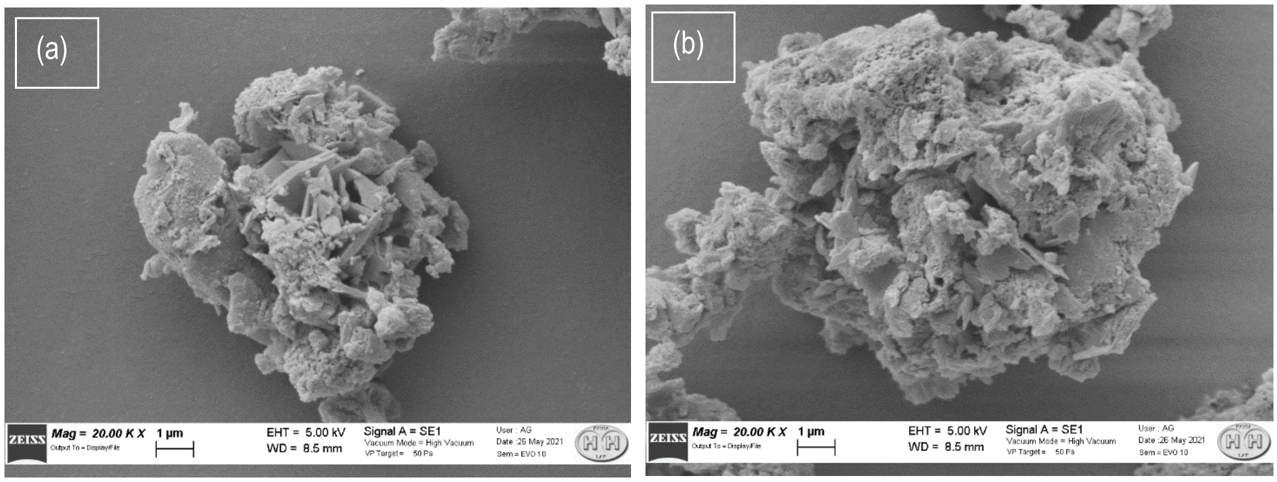

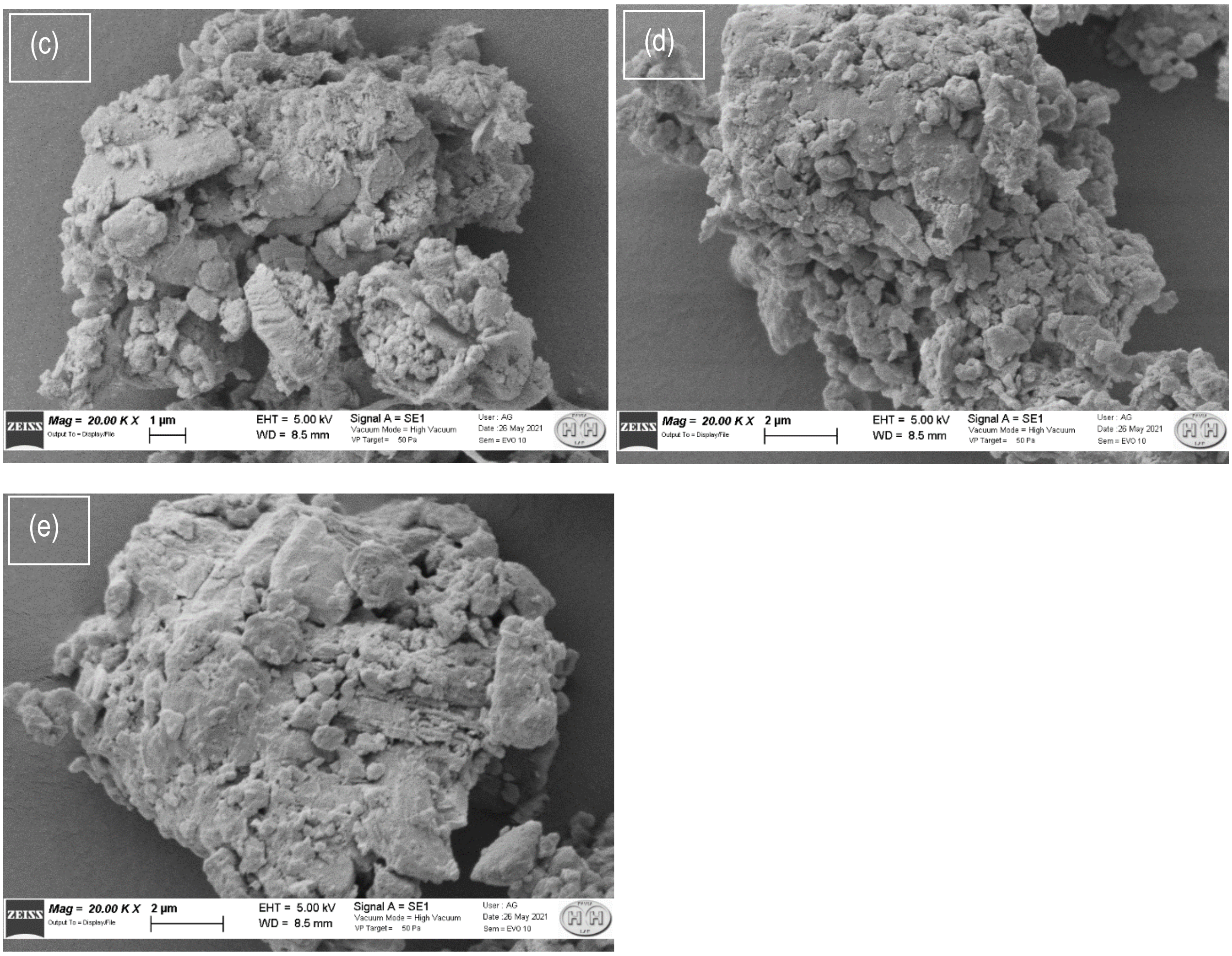

Figure S3. Scanning Electron Microscopy. SEM images of the $PhBz_2GeBr_4$/g-C_3N_4 composites at 1 (a), 2.5 (b), 2.5 (c), 5 (d), and 15 wt% of MHP.

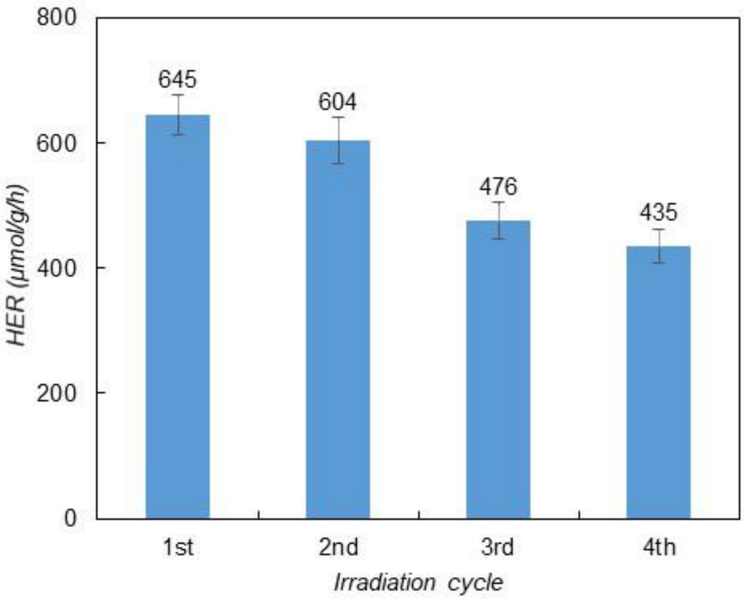

Figure S4. Cyclability test. Related to Figure 4. HERs after four successive photocatalytic cycles (6 h each) for $PhBz_2GeBr_4$ 2.5wt%/g-C_3N_4 (1 g L^{-1} catalyst, 3 wt% Pt, 10% v/v TEOA, simulated solar light (Xenon lamp, 500 W m^{-2}, 300-800 nm, IR-treated soda lime glass UV outdoor filter); RSD < 10% (*n*=3).

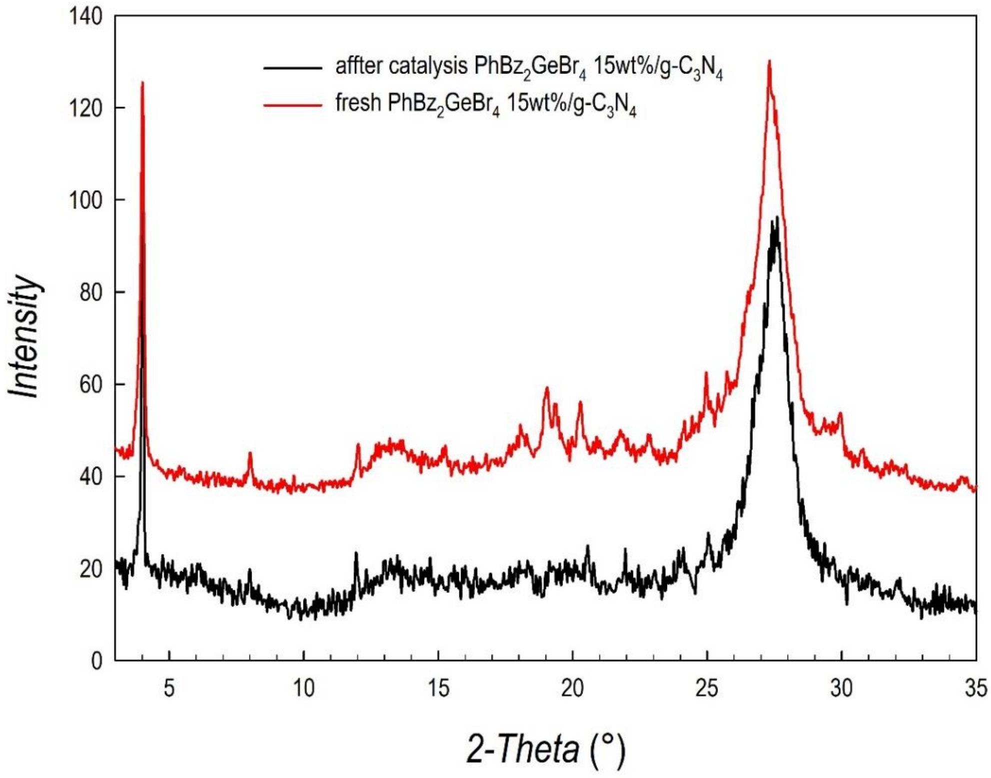

Figure S5. Post-catalysis Diffraction. Related to Figure 4. XRD pattern of 15 wt% $PhBz_2GeBr_4$/g-C_3N_4 before (red) and after (black) photocatalysis.

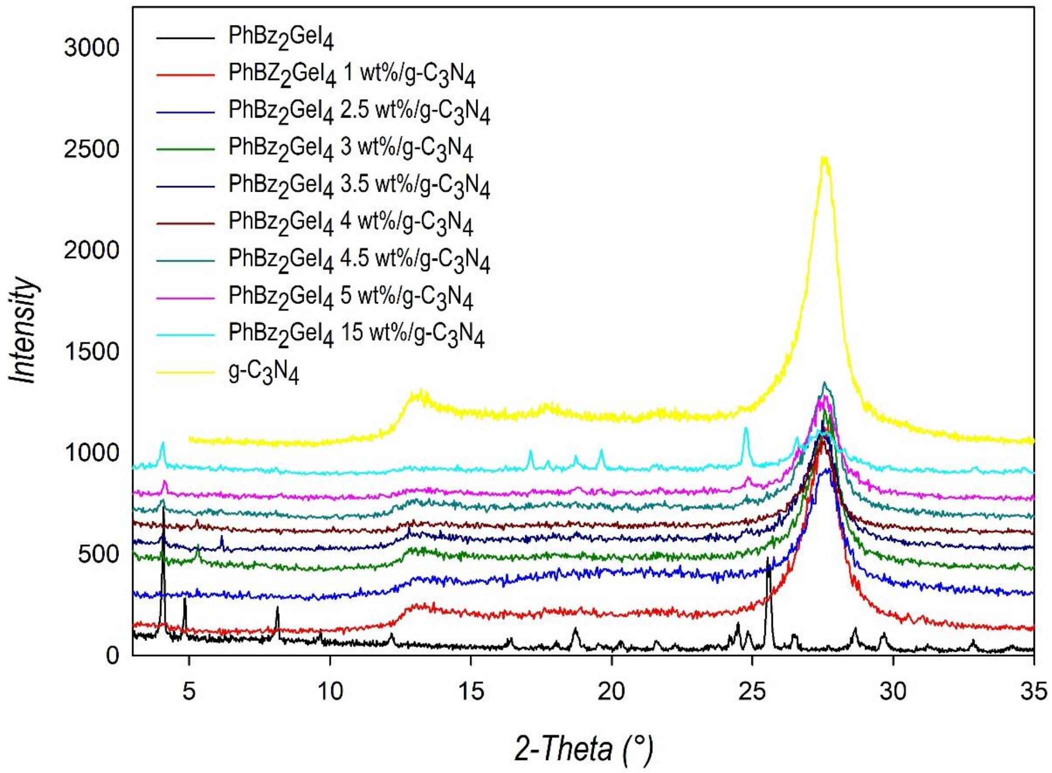

Figure S6. **Diffraction data of $PhBz_2GeI_4$/g-C_3N_4 composites.** Related to Figure 7. XRD patterns of $PhBz_2GeI_4$/g-C_3N_4 composites for different perovskite loadings

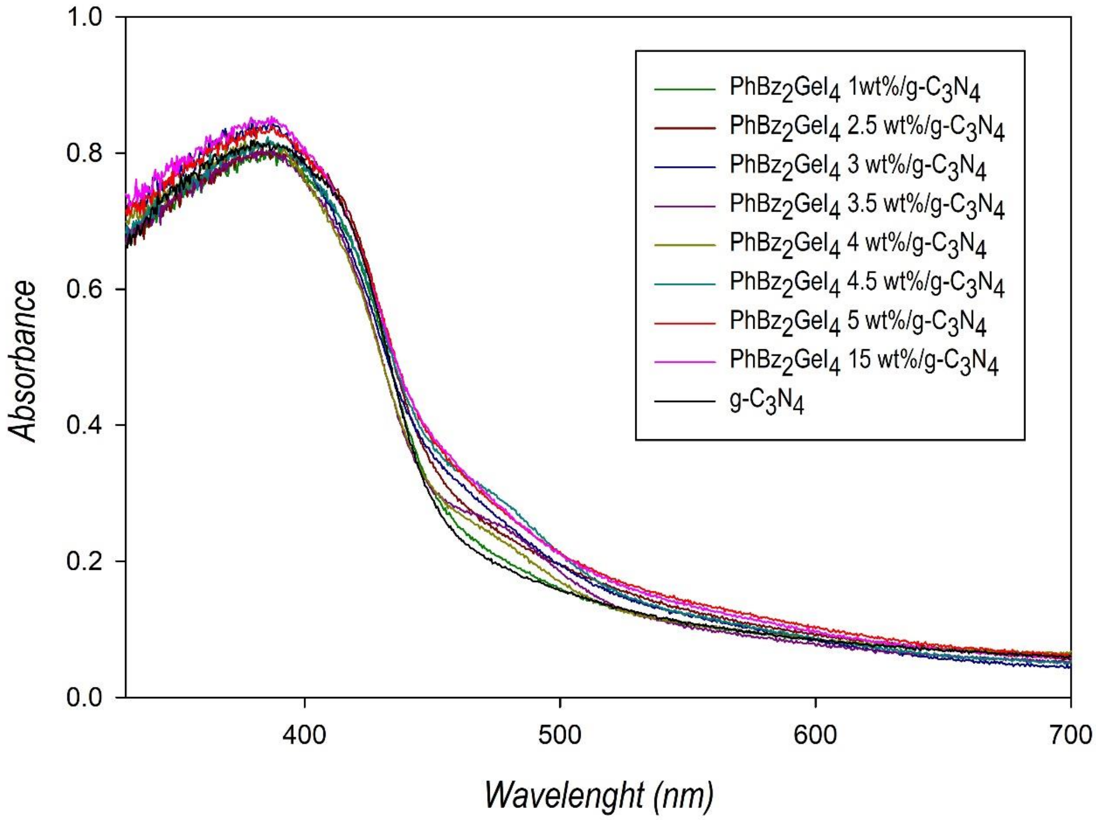

Figure S7. UV-Vis Spectroscopy of $PhBz_2GeI_4$/g-C_3N_4 composites. Related to Figure 7. UV-Vis spectra of $PhBz_2GeI_4$/g-C_3N_4 composites for different perovskite loadings.

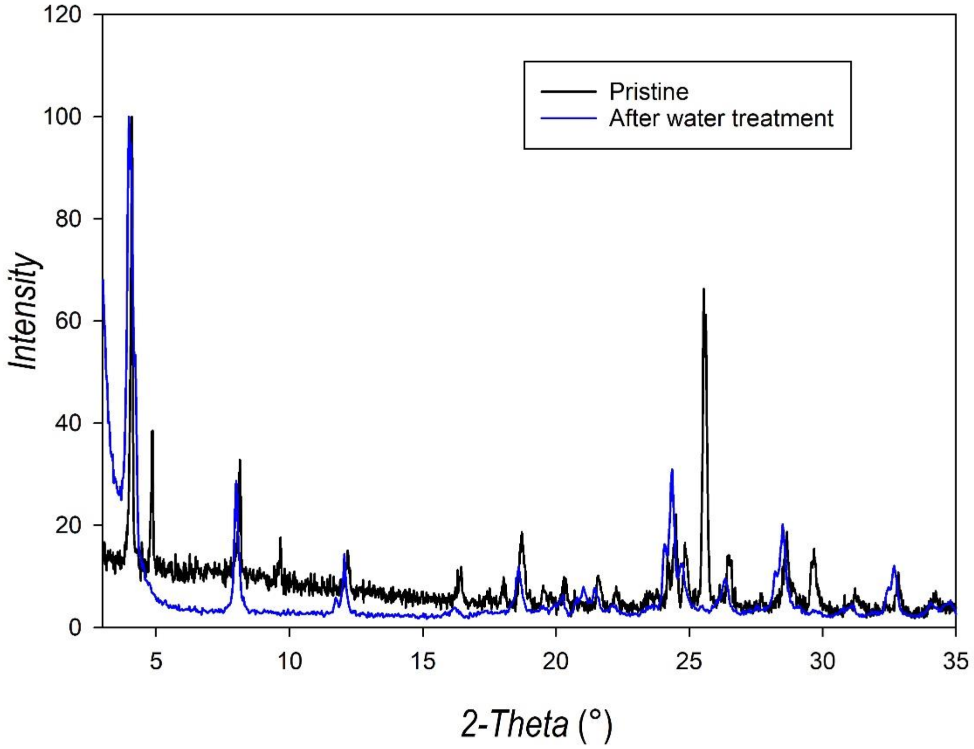

Figure S8. Diffraction after water-treatment. XRD pattern of $PhBz_2GeI_4$ before (black) and after (blue) water treatment.

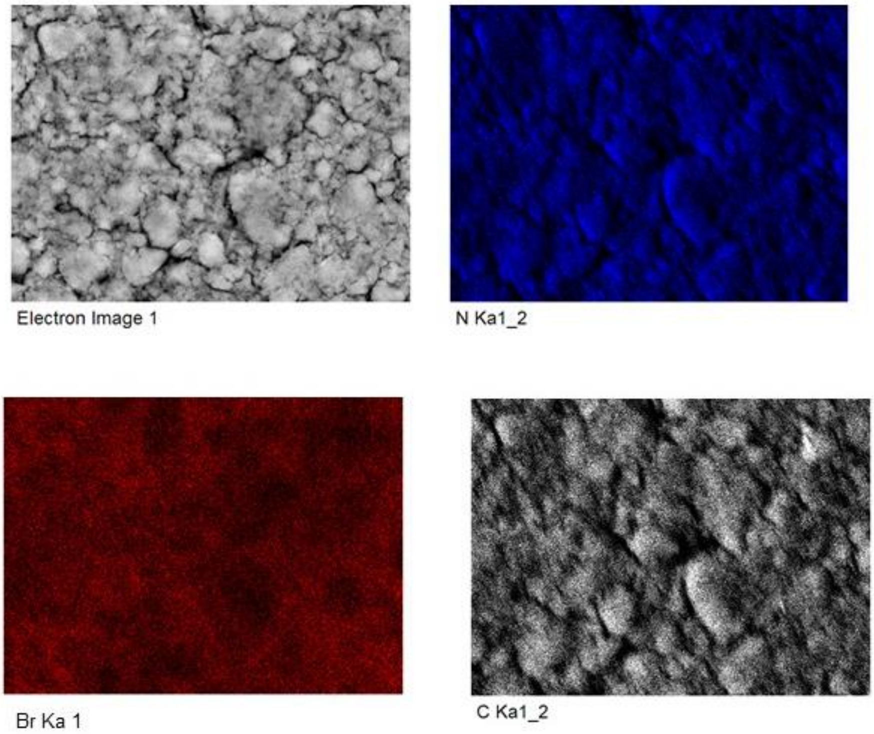

Figure S9. Elemental Mapping. Elemental mapping of $PhBz_2GeBr_4$ 2.5 wt%/g-C_3N_4 composite.

Table S1. Hydrogen Production Results with Biomasses. Related to Figure 4. HERs from model sacrificial biomasses.

	HER (μmol g^{-1} h^{-1})[a]	
	2.5% $PhBZ_2GeBr_4$/g-C_3N_4	3% $PhBz_2GeI_4$/g-C_3N_4
Glucose	55	151
Starch	35	78

[a]RSD < 10% (*n*=3). Conditions: 0.5 g L^{-1} catalyst, 0.5wt% Pt, 0.2 M glucose or 4.5 g L^{-1} starch, simulated solar light (Xenon lamp, 500 W m^{-2}, 300-800 nm, IR-treated soda lime glass UV outdoor filter), 6 h irradiation.

SUPPLEMENTAL NOTES

NOTE S1. Computational Details. Related to Figure 6.

Formation energies

All the calculations have been performed with the CP2K code.[1] Atom-centered Gaussian-type basis functions are used to describe the orbitals. We employ the MOLOPT[2] basis set and use a cutoff of 600 Ha for the plane waves. Core-valence interactions are described by Goedecker-Teter-Hutter pseudopotentials.[3]

To calculate the formation energies from first-principles as reported in Eq. 2 of the main text, we need to build atomistic models (i) of the considered A_2GeX_4 perovskites: $(PEA)_2GeBr_4$, $(BrPEA)_2GEBr_4$, $(BPEA)_2GeI_4$, and $(BPEA)_2GeBr_4$, X(ii) of GeX2 (X=I, Br) and, (iii) of AX. For $(PEA)_2GeBr_4$ and $(BrPEA)_2GEBr_4$, we construct supercells starting from the experimental crystallographic structures (cf. Table S2). Then, we perform density functional theory (DFT) calculations to relax both the coordinates of the atoms and the lattice parameters. These calculations are carried out employing the rVV10 functional, which accounts for van der Waals interactions and has been found to be suited to describe the energetics of layered and 2D materials.[4,5] In fact, the calculated lattice parameters for $(PEA)_2GeBr_4$ and $(BrPEA)_2GEBr_4$ nicely agree with those measured, with differences below 2%. To model $(BPEA)_2GeI_4$, we start from the experimental crystallographic structure of $(BPEA)_2PbI_4$[6] in which we replace Pb atoms with Ge atoms and then we fully relax both coordinates and lattice parameters. Analogously, we obtain an atomistic model of $(BPEA)_2PbBr_4$, by further replacing I with Br.

GeI_2 and $GeBr_2$ are analogously modelled constructing atomistic supercells from the experimentally available crystal structure: a=b=4.13 c=6.75, $\alpha=\beta=90^o$,$\gamma=120^o$ for GeI_2 and a=11.680, b=9.120, c=6.869 $\alpha=\gamma=90^o$, $\beta=101.9^o$ for GeBr2).[7,8] For GeI_2 ($GeBr_2$), we constructed a 4x4x3 (2x2x3) supercell. For AX salts, experimental data were not available for many of them. Therefore, we considered, as a reference, the total energy of an isolated AX dimer for all considered species.

Table S2. **Calculated structural parameters**. Related to Figure 6. Calculated values of the lattice parameters (Å) for the perovskites considered in this study (experimental values from Ref. 9 are given in parenthesis, where available) and number of atoms of the employed supercells.

Perovskite	Lattice parameters (Å)	Bond Angles (º)	Supercell	N. atoms
$(PEA)_2GeBr_4$	(11.406)	80.516	2x2x2	1312
	(11.524)	74.9498		
	(17.441)	90.012		
$(BrPEA)_2GEBr_4$	11.560 (11.396)	90	1x1x2	752
	11.515 (11.596)	90		
	72.810 (71.0480)	90		
$(BPEA)_2GeI_4$	44.565	90	1x2x2	1072
	16.929	90		
	17.360	90		
$(BPEA)_2GeBr_4$	46.911	90	1x2x2	1072
	15.878	90		
	16.623	90		

(100) surfaces of $(BPEA)_2GeX_4$ perovskites

We consider three possible symmetric surface terminations for $(BPEA)_2GeI_4$: (i) stoichiometric (s) (BPEA)I-terminated slab, (ii) non-stoichiometric (ns) (BPEA)I-terminated slab, ns-GeI_2-terminated slab (cf. Fig. S7). The details of the considered slabs are given in Table S3 and the respective structures are shown in Table S3.

For the s-(BPEA)I-terminated slab, the surface energy $\gamma(s)$ is simply given by:

$$\gamma(s) = \frac{E_{\text{slab}}(s) - nE_{\text{bulk}}}{2A}$$

where $E_{\text{slab}}(s)$ is the total energy of the stoichiometric slab, E_{bulk} is the total energy per formula unit of the bulk material, n the number of formula units in the slab supercell, and *A* is the surface area of the slab. For the non-stoichiometric slabs, we notice that these are generated when cleaving the surface along the plane perpendicular to apical Ge-Br bonds. Therefore, we first need to consider the cleavage energy E_{cl} which is defined as:

$$E_{cl} = \frac{1}{4}[E^{\text{v}}_{\text{slab}}(\text{ns1}) + E^{\text{v}}_{\text{slab}}(\text{ns2}) - \ nE_{\text{bulk}}]$$

where $[E^{\text{v}}_{\text{slab}}(\text{ns1})$ and $E^{\text{v}}_{\text{slab}}(\text{ns2})$ are the total energies of the slabs generated upon cleavage (i.e. without relaxation), while n is the number of formula units corresponding to the non-cleaved system. For each non-stoichiometric slab, the surface energy is then defined as:

$$\gamma(ns) = \frac{E_{cl} + E_{rel}}{A}$$

with:

$$E_{rel} = \frac{E_{\text{slab}}(\text{ns}) - E^{\text{v}}_{\text{slab}}(\text{ns})}{2},$$

where $E_{\text{slab}}(\text{ns})$ is the total-energy of the relaxed slab. Results calculated at the rVV10 level of theory are collected in Table S3 and clearly demonstrate that the s-(BPEA)I is the most stable termination. For this reason, we employ this termination also for both $(BPEA)_2GeI_4$ and $(BPEA)_2GeBr_4$ in the calculation of the band alignment.

Table S3. **Calculated structural parameters**. Related to Figure 6. Cell parameters (Å) for the (100) slabs considered in this study (for *z*, the size of the vacuum layer is given in parenthesis), number of atoms of the model, and surface energy γ (eV/Å^2).

Perovskite	Termination	Cell parameters (Å)	γ (eV/Å^2)
$(BPEA)_2GeI_4$	s-(BPEA)I	16.929, 17.360, 100.00 (~30)	0.005
	ns-(BPEA)I	16.929, 17.360, 100.00 (~35)	0.014
	ns-GeI_2	16.929, 17.360, 100.00 (~30)	0.015
$(BPEA)_2GeBr_4$	s-(BPEA)Br	15.878, 16.623, 100.00 (~30)	0.005

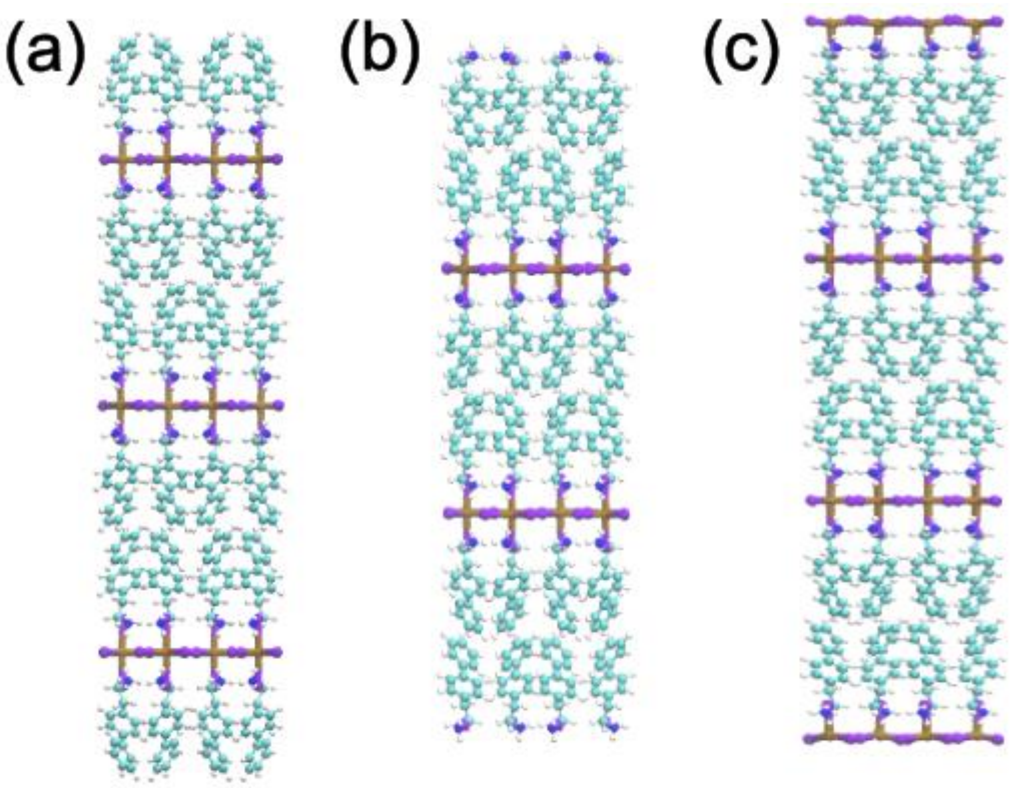

Figure S10. Calculated structural terminations. Related to Figure 6. Stick&ball representation (side view) of the different terminations considered for the (100) surface of $(BPEA)_2GeI_4$: (a) s-(BPEA)I, (b) ns-(BPEA)I, (c) ns-GeI_2. Ge atoms are depicted in ochre, I in violet, C in cyan, N in blue, and H in white.

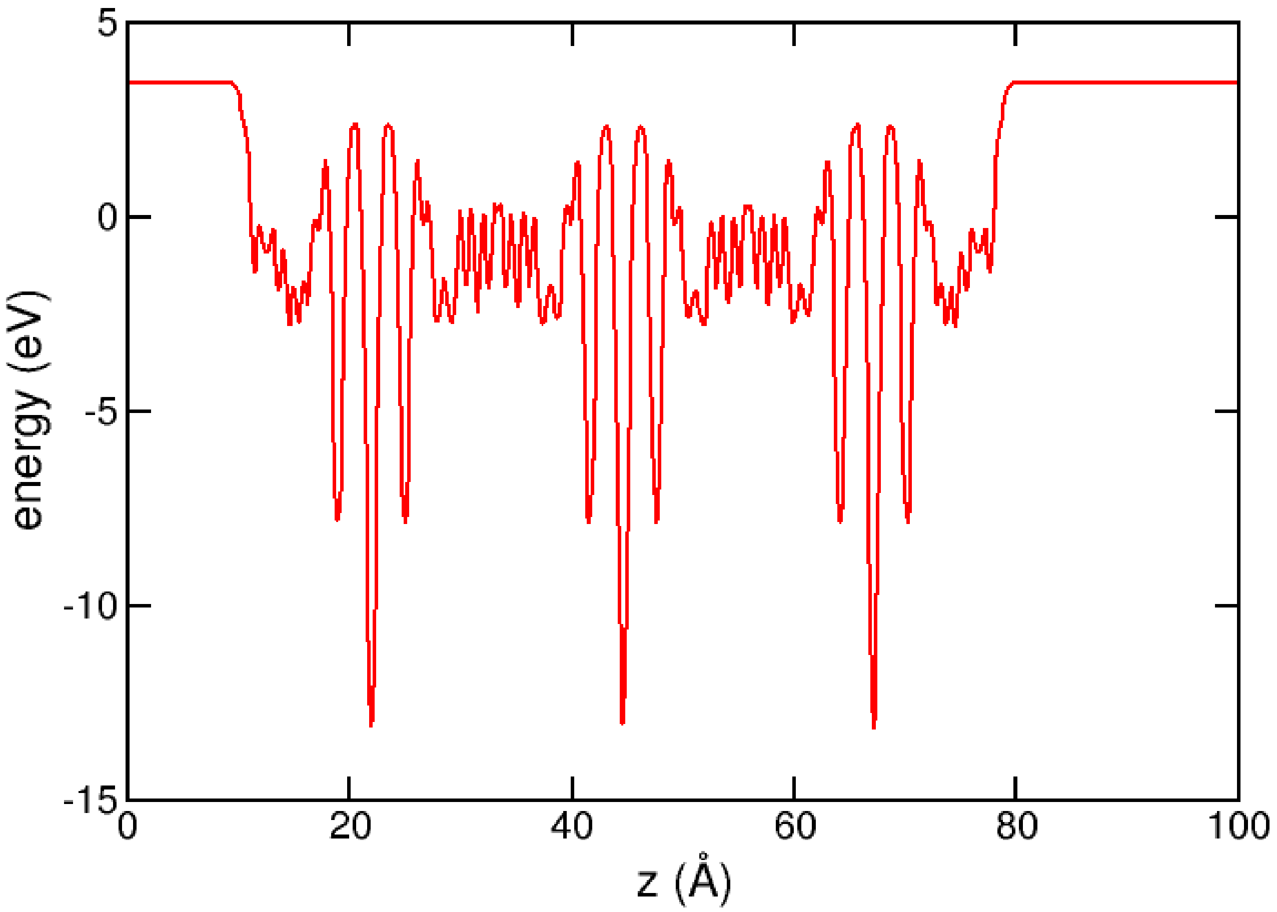

Figure S11. Electrostatic Potential. Related to Figure 6.v Average electrostatic potential referred to the vacuum level across the semiconductor-vacuum interface for $(BPEA)_2GeI_4$.

Band Alignment

We here employ electronic-structure calculations at the hybrid DFT level of theory, in order to align the band edges of the studied Ge-based perovskites with respect to the vacuum level. Hybrid-DFT calculations have been carried out with CP2K using the auxiliary density matrix method to speed up the calculation of exact exchange. To this end, we employ the cFIT auxiliary basis set.[10]

We note that the band alignment for g-C_3N_4 (reported in Fig. 7 of the main text) has been carried out in a previous study.[11] Therefore, we here report on the results achieved for $(BPEA)_2GeI_4$ and $(BPEA)_2GeBr_4$. First, we reproduce the experimental band gap of the perovskites by tuning the the fraction of Fock exchange α of the PBE0 functional [12,13] . This method has been found to produce ionization potentials, electron affinities and energy levels at the semiconductor-water interface in remarkable agreement with the experiment.[14,15] In fact, mean average errors of ~0.2 eV have been estimated for these quantities in screenings performed on a large set of semiconductors.[14]

The band edges are then aligned with respect to the vacuum level, by determining the electrostatic-potential line-ups across the surfaces (cf. Fig. S8). We note that the flat potential across the vacuum region ensures that no residual electrical field is present in the studied slabs (cf. Figure S7). Further, we position the standard hydrogen electrode (SHE) with respect to the vacuum level. In particular, we employ the theoretical alignment

presented in Ref. 13, which has been achieved combining molecular dynamics simulation of a water-vacuum interface with a computational hydrogen electrode.[16,17] The alignment scheme presented in Fig. 7 of the main text is completed including the measured TEOA/TEOA+ redox level.[18]

Solvation Gibbs free energies of A cations

We calculated the solvation Gibbs free energies $\Delta G^0_{\mathrm{solv}}$ of selected A-site cations (cf. Figure S9) in aqueous environment. This quantity is defined as:

$$\Delta G^0_{\mathrm{solv}}(\mathrm{A}^+) = G^0_{\mathrm{solv}}(\mathrm{A}^+) - G^0_{\mathrm{vac}}(\mathrm{A}^+)$$

where $G^0_{\mathrm{solv}}(\mathrm{A}^+)$ and $G^0_{\mathrm{vac}}(\mathrm{A}^+)$ are defined as the Gibbs free energy of the solute in aqueous solution and in vacuum. These quantities are calculated using the Gaussian09 program package.[19] In particular, we employ the B3LYP exchange–correlation functional,[20] with a 6-31++G** basis set for C N H and the ECP lanl2dz pseudopotential for Br and I.[21,22] The implicit effect of the solvent was included performing calculations with the conductor-like polarizable continuum model (C-PCM).[23] Furthermore, to account also for the solvent explicitly, we included three water molecules for each considered cations. Calculated results are reported in Table S4.

Table S4. Solvation Energies. Calculated values of $\Delta G^0_{solv}(A^+)$ (eV) for A-site cations employed in this work and in Ref. 7

**A-site cation**	$\boldsymbol{\Delta G^0_{\mathrm{solv}}(\mathrm{A}^+)}$
$BPEA^+$	-2.61
$PhBz^+$	-2.62
PEA^+	-2.80
$FPEA^+$	-2.84
BrPEA	-2.86
BzA^+	-2.92

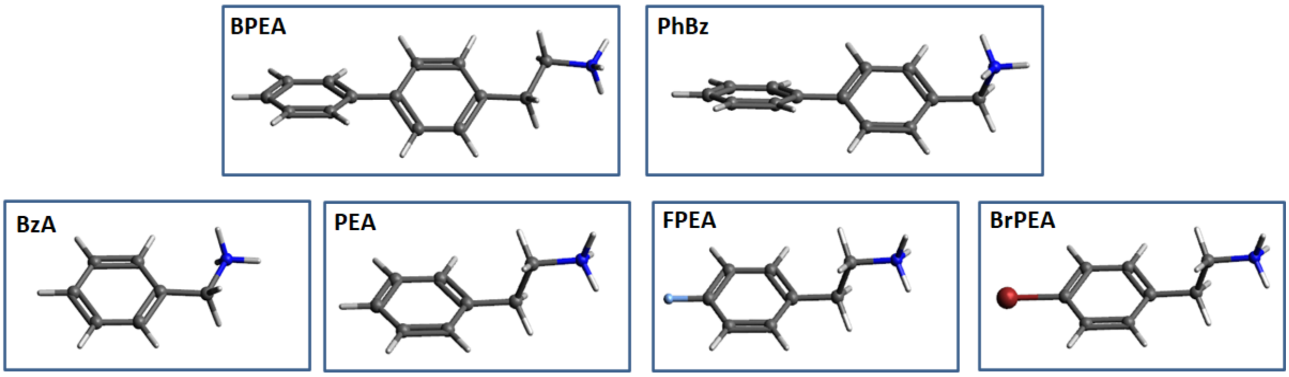

Figure S12. Cation structure representation for solvation calculations. A-site cations considered for the calculation of solvation Gibbs free energy in water. C in grey, N in blue, and H in white.

1. VandeVondele, J., Krack, M., Mohamed, F., Parrinello, M., Chassaing, T., and Hutter, J. (2005). Quickstep: Fast and accurate density functional calculations using a mixed Gaussian and plane waves approach. Comput. Phys. Commun. *167*, 103-128. https://doi.org/10.1016/j.cpc.2004.12.014.
2. VandeVondele, J., and Hutter, J. (2007). Gaussian basis sets for accurate calculations on molecular systems in gas and condensed phases. J. Chem. Phys. *127*, 114105. 10.1063/1.2770708.
3. Goedecker, S., Teter, M., and Hutter, J. (1996). Separable Dual-Space Gaussian Pseudopotentials. Phys. Rev. B *54*, 1703-1710. 10.1103/PhysRevB.54.1703.
4. Vydrov, O.A., and Van Voorhis, T. (2010). Nonlocal van der Waals density functional: The simpler the better. J. Chem. Phys. *133*, 244103. 10.1063/1.3521275.
5. Sabatini, R., Gorni, T., and de Gironcoli, S. (2013). Nonlocal van der Waals density functional made simple and efficient. Phys. Rev. B *87*, 041108. 10.1103/PhysRevB.87.041108.
6. Venkatesan, N.R., Mahdi, A., Barraza, B., Wu, G., Chabinyc, M.L., and Seshadri, R. (2019). Enhanced yield-mobility products in hybrid halide Ruddlesden–Popper compounds with aromatic ammonium spacers. Dalton Trans. *48*, 14019-14026. 10.1039/C9DT03074C.
7. Rouse, R.C., Peacor, D.R., and Maxim, B.R. (1977). The crystal structure of germanium dibromide. Z. Kristallogr. Krist. *145*, 161-171.
8. Powell, H.M., and Brewer, F.M. (1938). 35. The structure of germanous iodide. J. Am. Chem. Soc., 197-198.
9. Chiara, R., Morana, M., Boiocchi, M., Coduri, M., Striccoli, M., Fracassi, F., Listorti, A., Mahata, A., Quadrelli, P., Gaboardi, M., et al. (2021). Role of spacer cations and structural distortion in two-dimensional germanium halide perovskites. J. Mater. Chem. C *9*, 9899-9906. 10.1039/D1TC02394B.
10. Guidon, M., Hutter, J., and VandeVondele, J. (2010). Auxiliary Density Matrix Methods for Hartree−Fock Exchange Calculations. J. Chem. Theory Comput. *6*, 2348-2364. 10.1021/ct1002225.
11. Romani, L., Speltini, A., Ambrosio, F., Mosconi, E., Profumo, A., Marelli, M., Margadonna, S., Milella, A., Fracassi, F., Listorti, A., et al. (2021). Water-Stable DMASnBr3 Lead-Free Perovskite for Effective Solar-Driven Photocatalysis. Angew. Chem. Int. 60, 7, 3611-3618. https://doi.org/10.1002/anie.202007584.
12. Perdew, J.P., Ernzerhof, M., and Burke, K. (1996). Rationale for mixing exact exchange with density functional approximations. J. Chem. Phys. *105*, 9982-9985. 10.1063/1.472933.
13. Adamo, C., and Barone, V. (1999). Toward reliable density functional methods without adjustable parameters: The PBE0 model. J. Chem. Phys. *110*, 6158-6170. 10.1063/1.478522.
14. Guo, Z., Ambrosio, F., Chen, W., Gono, P., and Pasquarello, A. (2018). Alignment of Redox Levels at Semiconductor–Water Interfaces. Chem. Mater. *30*, 94-111. 10.1021/acs.chemmater.7b02619.
15. Ambrosio, F., Wiktor, J., and Pasquarello, A. (2018). pH-Dependent Catalytic Reaction Pathway for Water Splitting at the BiVO4–Water Interface from the Band Alignment. ACS Energy Lett. *3*, 829-834. 10.1021/acsenergylett.8b00104.
16. Ambrosio, F., Guo, Z., and Pasquarello, A. (2018). Absolute Energy Levels of Liquid Water. J. Phys. Chem. Lett. *9*, 3212-3216. 10.1021/acs.jpclett.8b00891.
17. Ambrosio, F., Miceli, G., and Pasquarello, A. (2015). Redox levels in aqueous solution: Effect of van der Waals interactions and hybrid functionals. J. Chem. Phys. *143*, 244508. 10.1063/1.4938189.
18. Sampaio, R.N., Grills, D.C., Polyansky, D.E., Szalda, D.J., and Fujita, E. (2020). Unexpected Roles of Triethanolamine in the Photochemical Reduction of CO2 to Formate by Ruthenium Complexes. J. Am. Chem. Soc. *142*, 2413-2428. 10.1021/jacs.9b11897.
19. Frisch, M.J. et al. (2009). Gaussian 09 . Gaussian, Inc., Wallingford CT, 2016.
20. Becke, A.D. (1993). Density-Functional Thermochemistry. III. The Role of Exact Exchange. J. Chem. Phys. *98*, 5648-5652. 10.1063/1.464913.
21. Ditchfield, R., Hehre, W.J., and Pople, J.A. (1971). Self-Consistent Molecular-Orbital Methods. IX. An Extended Gaussian-Type Basis for Molecular-Orbital Studies of Organic Molecules. J. Chem. Phys. *54*, 724-728. 10.1063/1.1674902.
22. Hay, P.J., and Wadt, W.R. (1985). Ab initio effective core potentials for molecular calculations. Potentials for K to Au including the outermost core orbitals. J. Chem. Phys. *82*, 299-310. 10.1063/1.448975.
23. Cossi, M., Rega, N., Scalmani, G., and Barone, V. (2003). Energies, structures, and electronic properties of molecules in solution with the C-PCM solvation model. J. Comput. Chem. *24*, 669-681. https://doi.org/10.1002/jcc.10189.
